# Assessment of Lipophilicity Descriptors of Selected NSAIDs Obtained at Different TLC Stationary Phases

**DOI:** 10.3390/pharmaceutics13040440

**Published:** 2021-03-24

**Authors:** Małgorzata Starek, Alina Plenis, Marta Zagrobelna, Monika Dąbrowska

**Affiliations:** 1 Department of Inorganic and Analytical Chemistry, Faculty of Pharmacy, Jagiellonian University Medical College, Medyczna 9, 30-688 Kraków, Poland; tryptofan56@gmail.com (M.Z.); monika.1.dabrowska@uj.edu.pl (M.D.); 2 Department of Pharmaceutical Chemistry, Medical University of Gdańsk, Hallera 107, 80-416 Gdańsk, Poland; aplenis@gumed.edu.pl

**Keywords:** lipophilicity, NSAIDs, thin-layer chromatography, chemometric methods, stationary phases

## Abstract

Lipophilicity study of selected NSAIDs, the group of the bioactive compounds usually used in humans and animals medicine, with the use of experimental and calculation methods was evaluated. LogP values are proposed and compared as descriptors of the lipophilicity of eleven compounds (from oxicams and coxibs). Obtained data were designated by thin-layer chromatography (TLC) in various chromatographic conditions, with stationary phases with different properties. The mobile phase systems were prepared by mixing the respective amounts of water and organic modifier, methanol and acetone, in the range of 30 to 80% (*v*/*v*) in 5% increments. Retention parameters (R_F_, R_M_ and R_M0_) were calculated and statistically evaluated to establish correlations. All experimentally determined R_M0_ values were compared with partition coefficients obtained by computational methods using linear regression analysis. Moreover, in order to extract information about the lipophilicity of compounds from large retention datasets, two chemometric approaches, namely principal component analysis (PCA) and cluster analysis (CA) were carried out. Established models of lipophilicity may have the potential to predict the biological activity of a number of drugs. The presented knowledge may also be of use during drug discovery processes, broadening the knowledge of potential ways to modify the physicochemical properties of chemical compounds.

## 1. Introduction

One of the important features of drug substances is their bioavailability, which determines the potential of the tested compound to cross biological membranes. In 1997, the WDI database (World Drug Index) was analyzed in order to determine the physicochemical properties responsible for the solubility and permeability of drugs through biological membranes. Lipophilicity has been found to be one of the most important descriptors determining cell barrier permeation [1,2,3]. Many diverse biochemical and pharmacological processes involved in drug action and the fate of compounds in the environment are known to be dependent on the lipophilic property of their molecules [4]. A wide variety of molecular parameters have been calculated and tentatively applied for the assessment of the relationship between biological activity and physicochemical characteristics [5].

Lipophilicity belongs to a basic physicochemical characteristic, which significantly determines the behavior of a molecule in a biphasic system. In biological systems, it largely determines the solubility of drugs in biological fluids, penetration through the biological membranes, rate of gastrointestinal drug absorption, affinity to plasma and tissue proteins and accumulation in the organism, etc. [6,7]. This property also affects the pharmacodynamics of the drugs and plays a key role in rational drug design, since lipophilicity is of primary importance in drug absorption and distribution [8]. Especially for substances whose target is the cell surface, lipophilicity should be determined in order to avoid undesirable diffusion through biological membranes into cells, individual cell organelles or the central nervous system. Therefore, information on the lipophilicity of substances is very helpful, especially in the first steps of designing new molecules with potential biological activity [9].

The currently used standard methods of drug administration, both orally and via injection, do not fully utilize their therapeutic potential. The main problem is the fact that the drug is distributed throughout the body, which reduces the chances of supplying the target site with the right dose, and requires the use of much higher initial doses of the substance. One of the goals of modern pharmacology is to improve pharmaceuticals so that they directly reach the disease site. Then, the dose of the taken drug could be reduced and the negative effects of its action on healthy tissues would be minimized [10]. Among the various ways to achieve this goal are, inter alia, the use of drug carriers, for example nanoparticles, that change the distribution method of drugs in the body and may enable, for example, target therapy via the creation of effective drug delivery systems [11]. Appropriate nanotransporters should have specific features that are necessary to achieve a given purpose, i.e., appropriate size, surface nature (hydrophilicity/hydrophobicity), biodegradability, etc. These features contribute to improvements in the bioavailability of drugs with poor water solubility and permeability through cell membranes. As mentioned above, lipophilicity of drugs is considered as a crucial physicochemical property which should also be taken into account in the nanotechnology that is utilized in drug discovery processes [12].

The issue of lipophilicity began to be discussed in the 19th century. In 1964, Hansch described the partition coefficient between the two phases of n-octanol: water (logP value for non-ionized substances) and presented it as a parameter of lipophilicity. Ionized substances are described by the distribution coefficient (D), defined as the ratio of the amount of non-ionized components in the oil phase to the amount of non-ionized and ionized components in the water phase at equilibrium [13]. Lipophilicity is a physicochemical property which describes the partition equilibrium of solute molecules between water and an immiscible organic solvent (octanol or saturated hydrocarbons) [14]. Water–octanol partition coefficients have been widely used in quantitative structure–activity relationships (QSAR) in medicinal and pharmaceutical chemistry [15]. Traditionally, it is expressed as the compound partition coefficient (P) between two immiscible liquids—an organic solvent (usually n-octanol or sometimes chloroform or alkanes) and water [16]: logP_o/w_ = logC_org_/C_w_, where C_org_ and C_w_ are concentrations of a neutral, monomeric form of the solute in the organic solvent and in water at the state of equilibrium. The logarithm of the n-octanol-water partition coefficient (logP) is considered a useful parameter in the study of the biological or pharmacological activity of structures [8,13].

The experimental methods for determination of lipophilicity have been classified into three groups: direct (shake-flask), indirect (chromatographic, spectrophotometric, optical, electrochemical, etc.) and calculation methods (specialized software using mathematical models) [17,18]. The conventional procedures are the “shake-flask” and “generator column” methods [19,20]. In these methods, the soluble concentration in each phase of the equilibrated water–immiscible organic mixture is determined by spectrophotometric or chromatographic methods. Such procedures are time-consuming, tedious, and limited in terms of pH range (−3 < logP < 4). Furthermore, they are intended to be used on extremely pure compounds [19,21]. Hence, nowadays, this complicated approach has been almost completely substituted by modern chromatographic techniques, mainly directed by adsorption and partitioning processes [22,23,24,25]. 

In 1941, it was demonstrated for the first time that the R_F_ value obtained by thin-layer chromatography (TLC) can be used to determine lipophilicity [26]. Research carried out by Martin and Synge confirmed that this value is correlated with the partition coefficient according to the equation: R_M_ = log[(1/R_F_) − 1]. The value of R_M_ depends linearly on the concentration of organic components in the mobile phase. Biagi et al. used RP-TLC for the first time to determine lipophilicity [27]. They also defined the parameter R_M0_ (the value of R_M_ extrapolated to zero organic modifier content in the mobile phase), considered as the most accurate measure of lipophilicity that can be used in the analysis of QSAR studies [28]. Chromatographic methods, especially TLC, require small amounts of compounds and they do not need to be very pure because their impurities are readily separated during the chromatographic process. TLC has many advantages, such as simplicity, low volumes of the mobile phase, low cost of analysis, repeatability, and the ability to distinguish the lipophilicity of molecules of similar structure. Contrary to HPLC, TLC is a fast and inexpensive technique that allows for the simultaneous analysis of compounds representing different chemical groups [29]. The most common separation techniques used for lipophilicity measurements are RP-HPLC and TLC on nonpolar stationary phases such as RP-18 or RP-8 [13,30,31,32] or, less frequently, normal-phase TLC on silica gel [33]. Other applicable stationary phases include CN, DIOL, NH_2_, cellulose, RP-2, aluminum oxide or cellulose coated with various oils (paraffin oil, silicon oil, plant oils). Additionally, rice starch has been used to measure the lipophilicity of a considerable number of molecules [34,35]. The relationship between obtained properties with biological activity has been assessed [36,37].

Correct selection of the stationary phase plays a very important role in the optimization of chromatographic systems [38,39]. The TLC plate coating can serve two purposes in the separation process. The surface of the coated material can made to contain chemical groups that actually interact directly with the solutes themselves and, thus, determines the extent of their retention and the selectivity of the phase system. In such case, the material acts as the stationary phase proper. Silica gel would be the classic example of such a material, the surface of which is formed by fused Si_6_O_6_ rings, characterized by a chair conformation [40,41]. The presence of hydroxyl groups renders the surface of silica gel highly polar. The analyte molecules can bind to the silica gel in two ways: through hydrogen bonds and dipole–dipole interactions, and the total force of the interaction is the sum of these two components. Due to the presence of functional groups on the surface of the silica gel, water molecules can also be absorbed. In this case, the adsorbent may be deactivated and used to separate polar compounds, e.g., alcohols, acids or amines. 

Nowadays, modifications of silica gel are much more important [40,41,42]. The gel as a carrier can be modified by adding chains of different sizes to certain silicon atoms with appropriate functional groups. NH_2_, CN, and DIOL-modified silica sorbents are less polar than conventional silica phases, making them ideal for separating hydrophilic or charged substances. Sometimes, due to the quite strong reactivity of the amino group, undesirable reactions may take place, e.g., the formation of Schiff bases. On the other hand, this reactivity may prove to be an advantage (optical isomers). Such compounds can easily be attached under mild conditions directly in reaction on TLC plates to generate chiral stationary PIKLE phases [43]. The cyano modification results in a little more hydrophobic character than the amino plate, with the following series of polarities: silica > DIOL > NH_2_ > CN > RP-2 > RP-8 > RP-18. Since it is less sensitive to the water content, the DIOL phase can be used as an alternative to silica in critical separation problems. 

Nonpolar adsorbents bind to the chromatographed substances by hydrophobic forces. Examples of such stationary phases are activated carbon and graphitized carbon black [44]. Modified nonpolar adsorbents of RP (reversed-phase) type include silica gels with bounded alkyl chains: C_2_, C_4_, C_8_, C_12_ and C_18_ [39]. The alkyl chains introduced into the silica gel make its surface hydrophobic. The most commonly used is the octadecylsilane C_18_ phase. The RP-8 phase is moderately polar and can be used for the preliminary separation of unknown compounds, including polar ones. Both can also be used in typical normal-phase systems [45]. In pharmacopoeias, the RP-2 phase is called “silanised silica” and can be used for the analysis of high molecular weight compounds [40].

Apart from the experimental methods, the lipophilicity can be estimated using software based on the different mathematical models [46,47]. Since measuring logP is still a time consuming process, computational methods are used instead, in particular, pre-screening for drug discovery. There are many computational variants for predicting this property, ranging from simple methods based on a small number of descriptors, to advanced neural network algorithms with thousands of correction factors. The reliability of computational methods decreases with increasing complexity of the structure. Basically, methods of calculation of logP are usually divided into two groups: the substructure and whole-molecule approaches [48,49]. The methods from the first class are based on dividing a solute structure into chemically meaningful fragments, and the logP value is evaluated on the basis of atom/fragment contributions. The second approach examines the whole molecule, and applies different molecular descriptors, e.g., molecular surface and volume, charge density, or topological indices. The most popular databases used to calculate the lipophilicity and predict the structure of a given compound are: AlogPs, ClogP, CS ChemDraw Ultra and others. Software such as Chem3D Ultra 8.0 calculate lipophilicity descriptors using fragmental and atomistic methods (e.g., LogPC—Crippen method, LogPB—Broto method) (www.cambridgesoft.com). Software such as Dragon Plus 5.4 calculate lipophilicity descriptors on the basis of topological descriptors (e.g., MLOGP1—Moriguchi method, ALOGP1—Ghose–Crippen method (www.talete.mi.it). By using ALOGPS 2.1-vcclab internet module some logP values have been derived (e.g., ALOGPs, AClogP, ALOGP, MLOGP, XLOGP2, XLOGP3) (www.vcclab.org). 

Non-steroidal anti-inflammatory drugs (NSAIDs) are widely used for the long-term treatment of chronic rheumatic diseases [50]. They have a broad spectrum of activity, which might suggest that the differences in the efficacy and tolerability of NSAIDs are in part due to variations in their physicochemical properties (e.g., pK_a_ ionization constants, solubility, partition coefficients) that establish their distribution in the body [51,52]. In terms of chemical structure, NSAIDs consist of hydrophilic acid (carboxylic acid, enol) and a lipophilic part (aromatic ring) and are quite strong acids (with pKa values between 3 and 5). Due to their acidic nature, they are already unionized in gastric juice. In the small intestine, there are also favorable conditions for the absorption of weak acids. Low values of the volume of distribution of NSAIDs in the tissues (0.1–1) indicate their low extravascular distribution. The high protein binding is due to their amphiphilic properties, which explains why they displace other drugs from protein binding [53]. In addition, the lipid-aqueous partition coefficients of NSAIDs provide an indication of their lipophilicity and ability to pass through membranes. The target enzyme of NSAIDs is cyclooxygenase (COX), the limiting enzyme of prostaglandin synthesis, which is localized to the endoplasmic reticulum and to a minor extent to the nuclear membrane [54]. Both the lipophilicity and promotion behavior of NSAIDs might be crucial molecular properties for COX activity. 

Numerous publications have dealt with the subject of determining the lipophilicity of various NSAIDs using chromatographic methods [6,7,25,55,56,57]. The obtained results have led to the conclusion that the RP-TLC method can be successfully used in research into the properties of NSAIDs [58,59]. Chromatographic data (R_M_ and R_M0_) were also compared with the calculated values of logP, and the obtained correlations indicate that this technique may be an appropriate choice for the lipophilicity determination of COX-2 inhibitors.

Increasingly, thanks to computerized multidimensional data analysis procedures, it is possible to extract systematic information, often dispersed in large data sets. Upon application of chemometric methods (e.g., principal component analysis (PCA), cluster analysis (CA)), the number of variables in a data set is reduced by finding linear combinations of the variables that explain most of the data variability [60,61,62]. PCA allows for a more objective and rational estimation and comparison of the determined lipophilicity. The scores corresponding to the first principal component appears to be one of the best solutions for the lipophilicity scale, resulting from retention data. In addition, a careful investigation of eigenvalues and eigenvectors (loadings) can offer useful information concerning the retention mechanism of the compounds [63,64].On the other hand, CA is a technique that consists of grouping similar observations into several clusters based on the observed values of several variables, the concept of which is similar to the discriminant analysis [65].

In a prior publication [25], we analyzed the logP values of various NSAIDs by RP-TLC. Experimentally obtained logP values showed differences depending on the environment (organic modifiers), based on the various mobile phases. As a continuation of our previous work on the investigation of the lipophilicity effect of chosen compounds, here, we report the behavior and lipophilic character of biologically active substances using stationary phases with different properties. The study involved eleven NSAIDs, including oxicams (piroxicam (P), meloxicam (M), tenoxicam (T), isoxicam (I)) and coxibs (celecoxib (C), etoricoxib (E), rofecoxib (R), valdecoxib (V), cimicoxib (CI), firocoxib (F), robenacoxib (RB)). We studied their chromatographic behavior by TLC using six various stationary phases: RP-18, RP-8, RP-2, silica gel modified by CN, DIOL and NH_2_. The main goal of this study was to compare the ability to predict lipophilicity on an example of substances selected by RP-TLC, modified NP-TLC and calculation data. In particular, the influence of stationary phases on the retention parameters was analyzed, and the experimental lipophilicity estimated by means of chromatographic indicators was compared with the theoretically calculated values of the partition coefficient obtained by computational methods using linear regression analysis. Additionally, CA and PCA analyses were performed to compare the logP values. 

## 2. Materials and Methods

### 2.1. Chemicals 

The compounds: piroxicam, tenoxicam, meloxicam, isoxicam, celecoxib, etoricoxib, rofecoxib, valdecoxib and firocoxib were obtained from Sigma-Aldrich. Cimicoxib and robenacoxib were extracted from Cimalgex tablets (Vetoquinol S.A., Lure, France) and Onsior tablets (Novartis Sante Animale S.A.S., Basingstoke, UK), respectively. Analytical grade methanol, acetone and water were purchased from Merck (Darmstadt, Germany).

### 2.2. TLC Analysis 

The chromatographic behavior of drugs was studied on stationary phases: TLC silica gel 60 RP-18 F_254_s (No 1.05559), TLC silica gel 60 RP-8 F_254_ (No 1.15424), TLC silica gel 60 RP-2 F_254_ (No 1. 16464), HPTLC silica gel 60 CN F_254_s (No 1.16464), HPTLC silica gel 60 DIOL F_254_s (No 1.12668), TLC silica gel 60 NH_2_ F_254_s (No 1.05533) plates, purchased from Merck. The standard solutions of compounds were prepared in methanol and applied in duplicate onto the plates by means of a 10 μL syringe (Hamilton Company, Bonaduz, Switzerland) in the form of 5 mm wide bands in increments of 5 mm. Chromatography was performed in a vertical developing chambers (Sigma-Aldrich, Laramie, WY, USA), which was saturated for 15 min at room temperature, using different proportion mixtures of Met/water or Ac/water (from 30 to 80%, in steps of 5%). The plates were developed for a distance of 9 cm in all cases. After development, the plates were dried in air at room temperature and examined under UV lamp (Camag, Muttenz, Switzerland) at 254 or 366 nm.

After developing, the retention parameter R_F_ values were calculated. Next, R_M_ values of each compound were obtained using the equation: R_M_ = log(1/R_F_ − 1). As R_M_ generally depends linearly on the concentration of the organic modifier in the mobile phase, in the next step, the values have been extrapolated to a zero concentration of organic component (to obtain R_M0_ values). The linear correlation between R_M_ values and the concentration of the organic modifier in the mobile phase were calculated separately for each compound by using the Soczewiński–Wachtemeister equation [31]: R_M_ = R_M0_ + aC, where a—the slope, C—the volume fraction of methanol or acetone in the mobile phase, R_M0_—the intercept (value related to the molecular lipophilicity). Obtained values were subjected to statistical evaluation and correlations between them were established by regression analysis. Next, experimental results were correlated with computational data.

### 2.3. Computational Calculations 

Various types of software are able to calculate lipophilicity values by different algorithms. All of them require a previous molecule drawing that is usually performed by Hyperchem and optimized using the MM+ molecular mechanics force field [66]. On the basis of the obtained geometry software, various lipophilicity descriptors can be calculated. In our work theoretical octanol/water partition coefficients were calculated by using the following algorithms: ALOGPs, AC_logP, ALOGP, MLOGP, XLOGP2, XLOGP3 offered from the Virtual Computational Chemistry Laboratory, and ChemAxon from DrugBank (www.drugbank.ca; www.vvclab.org; accessed on 20 May 2020). 

### 2.4. Chemometric Data Treatment 

Regression analysis for the establishment of the suitability of the tested chromatographic conditions and the validity of the computer programs was conducted by Statistica v.10 software (StatSoft, Tulsa, OK, USA). In these calculations, the correlation coefficients (r, r^2^), and the standard errors of the slope, interception and estimate (S_a_, S_b_, S_e_) were used as the basis for testing the linearity of regression plots. Principal component analysis (PCA) and cluster analysis (CA) were carried out using Statistica v.13.3 software (StatSoft, Tulsa, OK, USA). 

## 3. Results and Discussion 

In the described study, the retention parameters of NSAIDs belonging to the oxicam and coxib groups were analyzed. Molecular information about the tested analytes are presented Table S1 (Supplementary Materials). In the course of the research study, various types of stationary phases were used, differing in their chemical structure and physicochemical properties. Six different adsorbents (RP-18, RP-8, RP-2, CN, NH_2_, DIOL) were used, which are modifications of silica gel to which carbon chains of different lengths or functional groups were attached. After developing chromatographic plates with mobile phases consisting of methanol (Met)/water or acetone (Ac)/water mixtures, the spot positions were recorded and retention factors (R_F_) were calculated. The obtained R_F_ values depended, among other factors, on the chemical structure of the tested drugs. After studying the value of this parameter, it can be seen that it increases following an increase in the percentage of each of the organic modifiers in the mobile phase. 

In our investigations, a wide range of mobile phase organic modifier concentrations was used. For most substances, the R_F_ changes from 0.1 to 0.9 with Met and from 0.2 to 0.9 with Ac. For some of the more hydrophobic solutes, this range was somewhat narrower and mobile phases rich in an organic modifier were used. The highest R_F_ values were obtained on the plates with NH_2_ modified silica gel, and the lowest on RP-8. For RP-18, as the stationary phase, the R_F_ ranged from 0.01 to 0.82 for Met/water as the mobile phase and from 0.01 to 0.895 for Ac/water, whereas for CN plates, the R_F_ ranged from 0.01–0.89 for Met and 0.08–0.90 for Ac, and for DIOL—R_F_ values ranged from 0.14–0.91 for Met and 0.23–0.97 for Ac as the organic modifier.

The R_M_ value was the next parameter that was calculated and analyzed (Tables S2 and S3). It was found that the R_M_ values decreased with the increasing amount of organic modifier in the mobile phase in all cases. The highest R_M_ data were recorded on RP-18, and the lowest on the silica gel modified with DIOL and NH_2_ groups. 

Based on the linear dependence of R_M_ on the concentration of Met or Ac in the mobile phase for the tested compounds, the R_M0_ values were determined by extrapolation of the organic modifier in these systems to the zero concentration. Obtained values were subjected to statistical evaluation and a correlations were established (Tables S4 and S5). The obtained regression coefficients for all systems were usually higher than 0.94. Lower correlations may be associated with the various retention interactions that the studied stationary phases may exhibit. The determined values of the R_M0_ parameter, which is a measure of the lipophilicity of the substance, differ from one another. They depend on the chemical structure of individual compounds (the cyclicity of the molecule, length of the carbon chain, presence of specific groups of atoms or substituents), and also on the properties of the system (stationary and mobile phases). The higher the R_M0_ value, the greater the lipophilic character of the substance. R_M0_ values for the tested oxicams ranged from −0.2693 to 2.1593, while for coxibs, higher values ranging from −0.2205 to 4.4357 in Met/water and from −0.6517 to 3.7555 in Ac/water were obtained. The lowest R_M0_ values were obtained for I and M. The highest values of these parameters were observed for P. In the coxib group, the lowest R_M0_ was obtained for R and RB, and the highest for C. 

**Figure 1:**
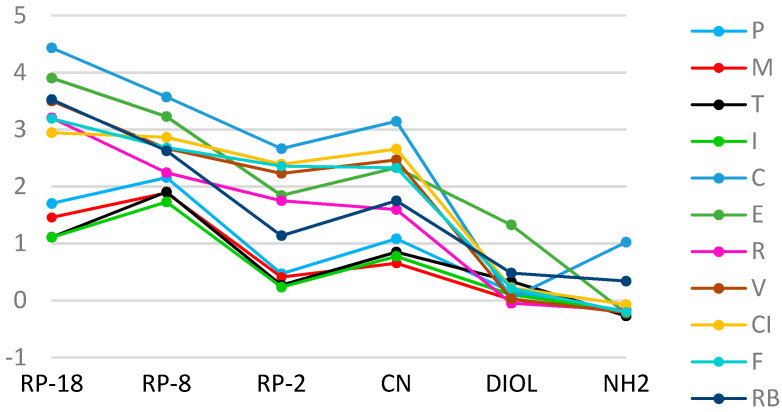
A plot of the relationship of R_M0_ values on the type of stationary phase for the tested substances in the methanol/water mobile phase.

**Figure 2:**
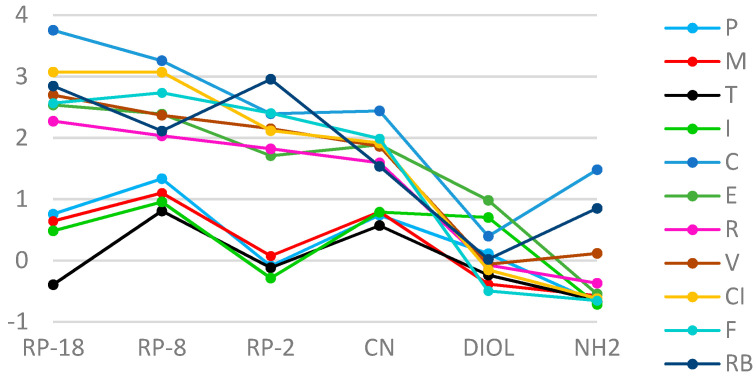
A plot of the relationship of R_M0_ values on the type of stationary phase for the tested substances in the acetone/water mobile phase.

From the comparative analysis of the linear correlation between R_M_ values of the tested compounds and the percentage concentration of organic modifiers, it can be concluded that the highest correlations were obtained for stationary phases with CN modified silica gel and RP-18 (r ≥ 0.98), and the lowest for RP-2 plates (0.82 ≥ r ≤ 0.99) in the Ac/water system. The best correlation was found on RP-18 plates for C (r ≈ 0.9983), and the lowest on CN plates for I (r ≈ 0.8296), and for T (r ≈ 0.8211) on RP-2. R_M0_ values obtained in the Ac/water mobile phase on RP and CN plates clearly separated into two groups, one—coxibs, and the second—oxicams. These differences can also be observed in the Met/water system, apparently for the analysis carried out on RP-18 plates. Based on these results, it can be concluded that, generally, I was the least lipophilic character, and C the greatest. Nonpolar stationary phases, such as RP-18 an RP-8, result in one achieving higher R_M0_ values compared to polar plates. When analyzing the dependence of R_M0_ on the stationary phase for each of the tested substances, a conclusion can be drawn: that the most similar values of this parameter were obtained on plates with gels modified with DIOL and NH_2_ groups. The greatest differences between R_M0_ values for individual NSAIDs were obtained on RP-18 plates (Figure 1 and Figure 2).

Taking into account the type of stationary phases used in both developing systems, it can be stated that the highest R_M0_ values for oxicams were found on RP-8, and for the coxibs on RP-18 (Table 1). The lowest R_M0_ values for both, oxicams and coxibs, were achieved on the stationary phases with the gel modified with NH_2_ groups. The R_M0_ determined on RP-18 plates in the Met/water mobile phase, ranged from 1.1091 (I) to 4.4357 (C), while on RP-8, they ranged from 1.7301 (I) to 3.5729 (C). In the Ac/water development system, the highest recorded R_M0_ value on RP-18 plates was 3.7555 (C), and the lowest was 0.3949 (T). On the RP-8 plates, the values of this parameter ranged from 0.8081 (T) to 3.2575 (C). In the case of NH_2_ modified silica gel, the values ranged—in both developing systems—from −0.7145 (I) to 1.4811 (C). These results confirm that the value of this parameter also depends on the structure and properties of the adsorbent used for the tests. 

When water and Met as an organic modifier were used, R_M0_ values were higher compared to those with water and Ac. However, in the Ac/water system, R_M0_ values were more varied. The obtained values indicate the weakest lipophilic properties among the oxicams, especially I, and the most potent was P. In the group of coxibs, R and RB showed the weakest lipophilic character, while the strongest one was C. The conducted analysis confirms that the application of RP-18 and RP-8 stationary phases, classified as nonpolar modified silica gel adsorbents with longer carbon chains, allows one to obtain higher values of the R_M0_ parameter compared to in the case of polar stationary phases. Moreover, coxibs show a much more lipophilic character than oxicams. This is especially evident in the analysis on RP and CN plates for both mobile phase systems. The smallest differences in R_M0_ results are observed on the silica gel modified with DIOL and NH_2_ groups.

**Figure 3:**
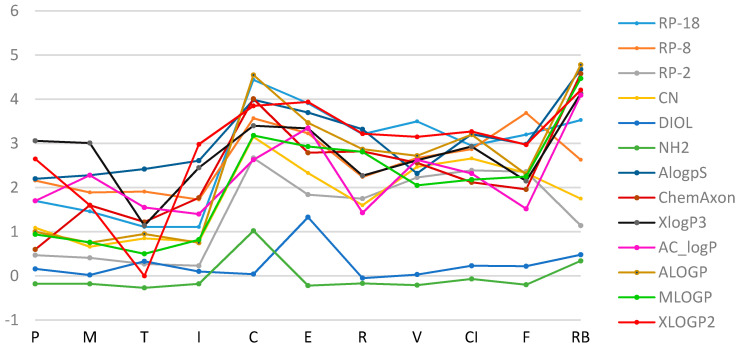
A plot of the relationship of R_M0_ values for the tested substances obtained experimentally (in the methanol/water mobile phase) and by calculation methods.

**Figure 4:**
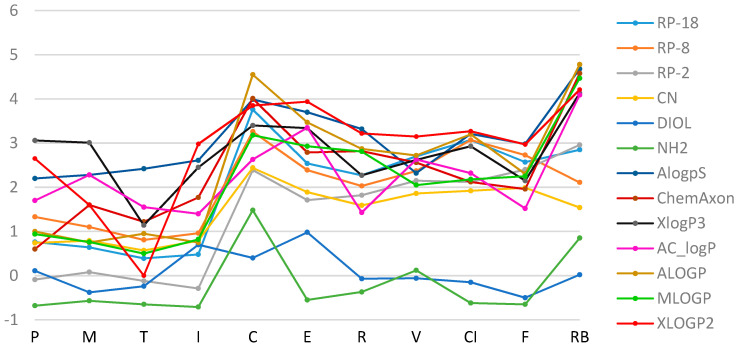
A plot of the relationship of R_M0_ values for the tested substances obtained experimentally (in the acetone/water mobile phase) and by calculation methods.

Apart from the experimental methods, the drug lipophilicity can be estimated using various chemical software products based on the different mathematical models. The experimentally obtained R_M0_ results were compared with the lipophilicity parameters calculated with various computer programs, based on the theoretical chemical structure of the analyzed molecules (ChemAxon, AlogPs, XlogP3, AC_logP, ALOGP, MLOGP, XLOGP2). It can be seen that logP_calc_ values, determined by theoretical calculations, differ depending on the type of software (Table 2). Generally, the most similar values of the experimental and calculated data were found on RP-18 chromatographic plates (Figure 3 and Figure 4). The most comparable logP data to the experimentally obtained R_M0_ of all studied NSAIDs were observed for AlogPs, ChemAxon and MLOGP. The largest differences between the experimental R_M0_ and calculated values were found on DIOL and NH_2_ modified silica gel plates.

In most cases, the calculated values are higher than those determined experimentally. The highest values of logP_calc_ were obtained using XlogP3, XlogP2 and AlogP, while the lowest were obtained experimentally, on silica gel plates modified with DIOL and NH_2_ groups (for both organic modifiers). The highest logP_calc_ values were obtained for RB and C, while the lowest values were obtained for all oxicams. A fairly large variation in logP_calc_ values was observed, especially for C and RB; the smallest differences were found with T. Moreover, for all coxibs, the results obtained on DIOL and NH_2_ plates ranged from −0.65 to 1.48, and differed from the other values obtained on RP and CN plates and by calculation methods (values in the range from 1.43 to 4.78).

Additionally, a linear regression analysis was applied to establish the suitability of the tested chromatographic conditions and the validity of the computer programs. Therefore, linear regression plots were calculated for obtained experimental R_M0_ values of the analytes studied, which were determined after using six tested adsorbents in Met/water and Ac/water system according to the equation: R_M0(1)_ = b + a R_M0(2)_. The detailed data for established linear regression plots, such as the values of a and b, the standard errors of the slope, interception and estimate (S_a_, S_b_, S_e_), as well as the correlation coefficients (r, r^2^), are reported in Table S6. Linear regression analysis was also performed for data sets describing the experimental R_M0_ of the tested substances calculated in both mobile phase systems and logP_cal_ parameters according to the equation: R_M0_ = b + a logP_calc_. A detailed description of the obtained results is shown in Table S7. Moreover, regression plots of logP_cal_ parameters calculated by seven different computer programs according to the equation: logP_cal(1)_ = b + a logP_calc(2)_ were also calculated, and these results are presented in Table S8. Table 3 shows the correlation coefficients (r) obtained for the linear correlation between the experimental R_M0_ values of the compounds of interest, established after using six different adsorbents in Met/water system (white area) and in Ac/water system (grey area). The correlation coefficients calculated between R_M0_ and logP_cal_ parameters, as well as for the analytes measured by the seven tested computer programs are also shown in Table 3. These data are presented in italics. These summarized data indicated that high correlations between the experimental R_M0_ parameters for the compounds of interest (r > 0.8375) were established between RP-18/RP-8, RP-18/RP-2, and RP-18/CN, independent of the type of organic modifier used in the mobile phase (Table 3 and Table S6). These correlation coefficients were lower when they were established in respect to NH_2_ (r from 0.4055 to 0.5601 and 0.5023 to 0.6500 for the Met/water and Ac/water system, respectively). The worst relationships of R_M0_ values calculated for the compounds of interest were found when they were compared to those established after using DIOL in both tested experimental conditions (from 0.0556 to 0.2770 and in the range of 0.0394–0.1266 for Met and Ac, respectively). Taking into account the correlation obtained between the experimental R_M0_ values of the analytes measured after using Met as the organic modifier and logP_cal_ parameters, these values were higher than 0.7094 when RP-18/AlogPs, RP-18/ChemAxon, RP-18-MLOGP, RP-18/XLOGP2, RP-2/ALOGP, CN/ALOGP and NH_2_/ChemAxon were used. When the experiments were performed using Ac, the correlation for AlogPs compared to RP-18, RP-2; ChemAxon with RP-18, RP-2 and NH_2_, as well as ALOGP with RP-18, RP-8, RP-2, CN, and NH_2_, as well as MLOGP with RP-18, RP-2 and CN, also including XLOGP2/RP-18 and XLOGP2/CN was in the range of 0.7086–0.9213 (Table 3 and Table S7). High relationships were found for lipophilicities established by the computer programs, including AlogPs with ChemAxon, ALOGP, MLOGP and XLOGP2 (from 0.8431 to 0.9251), ChemAxon with ALOGP (0.9136) and MLOGP (0.9183) and between ALOGP and MLOGP (0.9562) or XLOGP2 (0.8678), as well as MLOGP with XLOGP2 (0.8583) (Table 3 and Table S8).

For a more detailed interpretation of the range of experimental lipophilicity results, two statistical techniques, PCA and CA were used. PCA uses an orthogonal transformation for converting a data set of observations of potentially correlated variables into a set of values of linearly uncorrelated variables called principal components (PCs) [67,68]. This means that PCA transforms the original measured data into new uncorrelated (independent) variables called PCs, which are a linear combination of the original variables. These PCs are arranged in order of decreasing variance and create the basis of the respective vector space. In effect, two or three PCs ensure a good summary of all the variables, which can be used for testing the relationships between the objects (e.g., tested active substances) and the variables (e.g., data sets describing lipophilicity) by finding trends, groupings or outliers on loadings and score plots. Consequently, the chromatographic behavior of the tested compounds and the mechanism of their retention can be more precisely described [62,69]. Moreover, the adaptation of the first score of PCA (PC1), calculated straightway from the R_M0_ values, corresponding to an experimentally used organic modifier, allows for creating a new scale of lipophilicity [30]. In the current study, this new scale of lipophilicity was also used. Therefore, a scaled PCA was calculated for the retention parameters (R_M0_ values) of two different organic mobile phase modifiers (Ac and Met) and six used adsorbents: RP-2, RP-8, RP-18, DIOL, CN and NH_2_. Next, the first PC1_RM0 was used as a new parameter of lipophilicity, which was compared to the profiles of lipophilicity indicate by the seven computational methods, 12 values of the raw experimental R_M0_ measured using Ac or Met and six tested adsorbents (Table 2), as well as 12 values of the slope (a) which were calculated separately for each compound using the Soczewiński–Wachtmeister equation (Tables S4 and S5). The last parameter evaluates the rate at which the solubility of the solute increases in the mobile phase and it is considered an alternative measure of lipophilicity. The value of “a” is associated with the specific hydrophobic surface area of the molecule which plays an important function in the biological activity of the substance. This phenomenon was confirmed by the “r” and “a” correlation [70,71]. However, so far, this parameter has not been compared by multivariate methods in respect to other indicators of lipophilicity. Therefore, the whole dataset of dimensions—11 compounds × 32 lipophilicity measures—was subjected to another scaled PCA to evaluate the multivariate similarity. 

**Figure 5:**
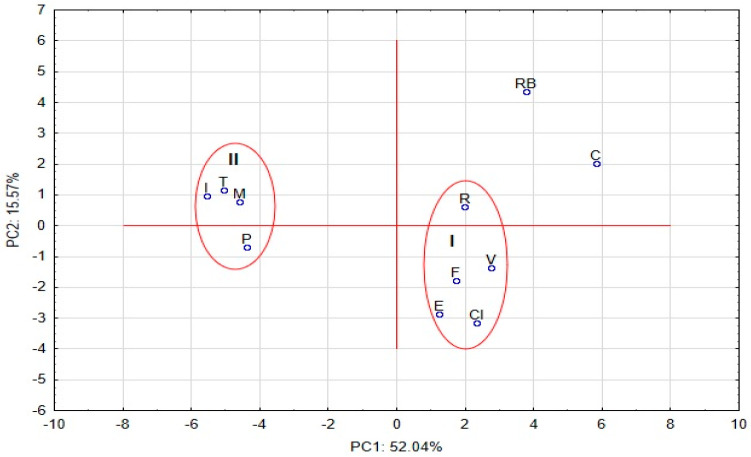
The score plots based on the autoscaled lipophicility results for analyzed compounds, studied picturing the objects in a two-dimensional space.

**Figure 6:**
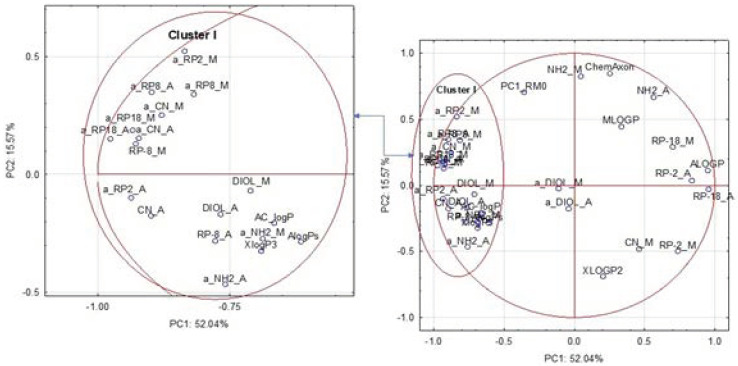
The loadings principal component (PC) plots based on the autoscaled lipophicility results for the studied compounds picturing the variables in two-dimensional space.

The score and loading plots based on the autoscaled lipophilicity results for eleven studied compounds picturing the objects and the variables in two-dimensional space are presented in Figure 5 and Figure 6, respectively. The localizations of the tested substances and the variables on the PC1 axes were mainly related to the variability of RP-8_M, RP-18_A, CN_A, ALOGP, and the parameters of “a”, calculated for RP-18, RP-8, RP-2 and CN, independent of the organic modifier used for the mobile phase. The variance of the analyzed data explored by the PC2 was mainly related to the variability of PC1_RM0, NH2_M, NH2_A, ChemAxon and XLOGP2. These two PCs explain more than 67.61% of the data variability. 

The graphical data presented in the score PC plot indicate that the positions of the compounds were visibly correlated with their chemical structures (Figure 5). Therefore, R, F, V, E and CI, belonging to the coxib group were included in cluster I. These compounds have sulfonylamide (-SO_2_NH_2_) (V, CI) or metylsulfonyl (-SO_2_CH_3_) groups (R, E, F). The coxibs with halogen atoms (three fluoride atoms in the side chain of pyrazol for C and four fluoride atoms in phenyl substitute for RB) were positioned as the outliers. Additionally, RB, in respect to the other tested coxibs, has a carboxyl group, while there is a lack of sulfonamide or metylsulfonyl substitute. This indicates that the presence of -SO_2_NH_2_ or -SO_2_CH_3_ or the acetic acid structure in the specific localization of the coxib molecule can determine the final interaction between the analyte and the molecules of the solvents used as the mobile-/solid-phase components, depending on the specific experimental conditions. However, these interactions can also be modified by fluoride atoms. In fact, higher lipophilicity parameters were calculated for C in most TLC conditions, as well as both C and RB by computational methods, than for the other coxibs included in cluster I.

All analytes included in cluster II belong to the oxicam group, with 1,2-benzothiazine-3-carboxamide 1,1-dioxide (P, M and I) or thieno[1,2]thiazin-4-one 1,1-dioxide (T) as the specific structure of these molecules. This structure determines the final interactions between the oxicams studied and the components of the mobile phase, as well as the molecules of the solid phases used in specific experimental conditions. The substitutes, such as the pyridinyl group (T, P), 5-methyl-2-thiazolyl group (M) and 5-methyl-1,2-oxazolyl group (I), probably have an inconsiderable influence on the chromatographic behavior. These observations were correlated with the numerical data summarized in Table 2, where the values of R_M0_ were relatively comparable for the selected adsorbent in specific TLC conditions, but they were variable depending on the use of the stationary phase and Met/water or Ac/water system. Moreover, most logP parameters for oxicams calculated by computational methods were higher than those obtained using TLC, but these values were similar taking into account the selected computational approaches.

The loadings PC plot based on the autoscaled lipophilicity results for the studied compounds is shown in Figure 6. It indicates that most parameters associated with the specific hydrophobic surface area of the molecule were positioned in cluster I, located on the left of the plot. In this cluster, there are also R_M0_ values calculated after using RP-8 and DIOL stationary phases, independent of the use of the type of organic mobile phase component, as well as the experimental logP values obtained on the CN stationary phase with Ac. Additionally, three computational methods including ALOGPs, XlogP3 and AC_logP were positioned in this cluster. This indicates that the profiles of the lipophilicity, expressed by the slope values calculated for the tested compounds on the basis of the Soczewiński–Wachtmeister equation, are comparable to those expressed by R_M0_ and logP values which were calculated by the methods included in cluster I. It may also suggest that these approaches are probably present in the most representative way the specific hydrophobic surface area of the molecule, which plays an important role in biological activity of the substance. Only a_DIOL M and a_DIOL_A parameters were positioned together in the middle of the score plot as the outliers in respect to the other experimental and calculated logP values located on the right of the plot. This may indicate that their values can be treated as a compromise parameter for describing both the specific hydrophobic surface area of the molecule and the lipophilic character of the compounds explained by the R_M0_ values measured on RP-2 and RP-18 adsorbents with the Ac/water and Met/water system, CN with Met and the calculated logP provided by XLOGP2 and ALOGP. It can also be observed, that the localizations of R_M0_ and the calculated logP parameters in the right and upper part of the plot, also including the PC1_RM0 calculated for the scaled R_M0_ established in 12 tested experimental conditions, were more varied than those observed for the approaches included in cluster I. Thus, the first PC1_RM0 was positioned between cluster I and logP, calculated by ChemAxon and MLOGP, as well as the R_M0_ obtained for the NH_2_ adsorbent, independent of the organic mobile phase component used. This may indicate that this parameter differences the tested compounds, representing a compromise between the specific hydrophobic surface area of the molecule, and the experimental logP in the specific TLC conditions and that calculated by ChemAxon and MLOGP approaches. On the other hand, the localizations of the R_M0_ parameters measured using the RP-2 and RP-18 adsorbents, independent of the organic modifier of the mobile phase and CN_M, were in close proximity to the positions of the logP established by ALOGP and XLOGP2. This may suggest that the differences in the lipophilicity profiles of the compounds studied calculated by these approaches were similar.

**Figure 7:**
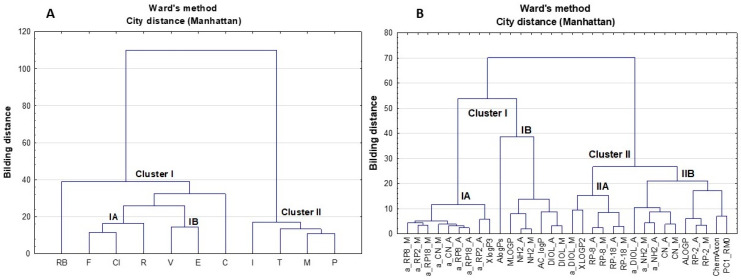
Dendrograms calculated on the basis of the established lipophilicity results for the studied substances using CA approach ((**A**) objects, (**B**) variables).

Next, hierarchical CA was performed for the same data set, describing the lipophilicity of the tested compounds. This approach allows one to find relatively homogeneous clusters of cases based on dissimilarities or distances between objects. The analysis starts with each case as a separate cluster (i.e., there are as many clusters as cases), and then combines the clusters sequentially, reducing the number of clusters at each step until only one cluster is left [72]. In consequence, the clusters are linked at increasing levels of dissimilarity in the form of a hierarchical tree diagram or dendrogram, which presents the hierarchical relationships in the tested data set. In this study, Ward’s distance was used for measuring the dissimilarity between each pair of observations, while city distance (Manhattan) was applied to determine which clusters should be joined at each stage. Dendrograms calculated on the basis of the established lipophilicity results for the eleven substances studied are illustrated in Figure 7A (the objects) and Figure 7B (the variables), respectively. It should be noted, that the CA results indicate comparable relationships between objects and the variables in respect to those found by the PCA. Thus, all coxibs were included in cluster I, where RB and C were located in reasonable proximity to other analytes (Figure 7A). As mentioned above, V, CI and C have a sulfonylamide group, while R, E and F possess a metylsulfonyl substituent. These components probably determine the final lipophilicity of the whole molecule. The presence of the carboxyl group in RB and fluoride substituents in RB and C allows for obtaining slightly different lipophilicity profiles to those calculated for the other coxibs studied. This was probably dependent on heterogenic atoms with free electron pairs, which created other interactions during chromatographic separation. Moreover, the CA results also grouped all of the studied oxicams in one cluster (II) (Figure 7A), as was previously observed in the score PC plot presented in Figure 5. Thus, the CA approach also indicates that the dominant influence on the behavior of the tested oxicams in TLC conditions is probably due to the specific structure of this class of NSAIDs, whereas the substitutes included in the molecules were not able to significantly alter the interaction that occurs during chromatographic separation.

Comparable to the PCA results, the CA plot for the variables illustrated in Figure 7B also shows that most parameters associated with the specific hydrophobic surface area of the molecule (a) were localized on the left of the dendogram together with the lipophilicity profiles calculated by XlogP3 (subcluster IA). The advantage of the CA approach is the fact that the localization of a-values in the dendogram clearly confirms that these parameters were mainly correlated with the organic solvent used for preparing the mobile phase, rather than with the physicochemical properties of the tested stationary phases which were used in the TLC conditions. Moreover, CA grouped the R_M0_ values calculated on DIOL and NH_2_ adsorbents—independent of the use of the organic mobile phase component—together with the logP parameters calculated by AlogPs, MLOGP and AC_logP in subcluster IB. This means that the two multivariate approaches used illustrated the relationships between the variables in slightly different ways. As mentioned earlier, the PCA positioned the experimental R_M0_ calculated on NH_2_ and MLOGP close to the PC1_RMo and ChemAxon parameters. On the other hand, both chemometric analyses confirmed that the logP parameters calculated by MLOGP gave comparable differences in the lipophilicity profiles established after using the NH_2_ adsorbent.

Moreover, CA grouped the logP calculated by XLOGP2, the parameters of a_DIOL and a_NH2 and R_M0_ measured on RP-8, RP-18 adsorbents—independent of the organic mobile phase used—in subcluster IIA. This suggests that these parameters comparably differentiate the tested compounds. Additionally, the lipophilicity expressed by R_M0_ for RP-8 and RP-18 stationary phases was mainly dependent on the adsorbent used rather than the organic modifier of the mobile phase. The same observation can be seen for the R_M0_ parameters calculated after using the CN adsorbent, which were included in subcluster IIB by CA, together with a_NH2 parameters. In subcluster IIB, ALOGP and the R_M0_ measured using the RP-2 adsorbent with Ac and Met as organic mobile phase modifiers, together with the first PC1_RM0 and ChemAxon were also positioned in close proximity. These localizations were comparable to those observed in the loading PC plot. Additionally, the localization of R_M0_ for RP-2 in the CA plot also confirms that the lipophilicity profiles of the compounds studied expressed by these parameters were mainly dependent on the adsorbent used rather than the organic modifier of the mobile phase. These data are not as clearly presented in the loading PC plot (Figure 6).

The chromatographic behavior of the compounds on the same type of TLC plates used in this study is similar to and in a very good agreement with their polarity, as can be easily observed in the table of correlations. These good regularities were also found by applying a scaled PCA and CA directly to the R_M0_ values matrix, as well as the parameters calculated on the basis of the Soczewiński–Wachtmeister equation, which are associated with the specific hydrophobic surface area of the molecule. These findings might indicate that the same mechanism (lipophilic interactions) is dominant in all cases.

## 4. Conclusions

In the present study, the retention parameters of the selected NSAIDs were determined using TLC with various stationary phases. Drugs from the oxicam and coxib groups were included in the research. Stationary phases (RP-2, RP-8, RP-18, silica gel modified with NH_2_, CN and DIOL groups, respectively) differing in their chemical properties were used, as well as water developing systems containing a variable percentage of methanol or acetone as organic modifiers. The experimental values of the lipophilicity parameters of the tested substances (R_M_ and R_M0_) were compared with data obtained by calculation methods.

Through analyzing the results, it was found that the lowest R_M0_ values were determined for isoxicam and the largest for celecoxib. In addition, the use of nonpolar RP-18 and RP-8 stationary phases resulted in higher R_M0_ values being obtained than in the case of polar adsorbents. The NSAIDs included in the study plan can be ranked according to their increasing lipophilic properties: I < T < M < P < R < F < V < E < CI < RB < C. The R_M0_ values obtained by linear extrapolation of the plots of R_F_ versus the concentration of the organic modifiers to its zero concentration are a good measure of the lipophilicity of low-to-medium lipophilicity compounds when methanol is used. However, acetone makes it possible to evaluate the logP versus that of compounds with higher lipophilicity that cannot be assessed with methanol.

In the study, the chromatographic data were compared with theoretical partition coefficient values calculated using computer programs, using regression analysis. Additional information about the relationships between the lipophilicity profiles of the oxicams and coxibs, calculated using both experimental and computational approaches, were obtained by multivariate tools such as PCA and CA. These chemometric methods correctly distinguished the substances studied and indicated the relationships between the chemical stucture of the analytes and lipophicility profiles. PCA and CA both resulted in consistent classification of the objects and comparable results for the variables described by the values of R_M0_, the parametrs of a, the first PC1 and calculated by logP. Moreover, CA more clearly indicated that the lipophilicity expressed by R_M0_ was probably mainly dependent on using the adsorbent rather than the organic modifier of the mobile phase. To the contrary, a-values associated with the specific hydrophobic surface area of the molecule were mainly correlated with the organic solvent used for preparing the mobile phase rather than the physicochemical properties of the tested stationary phases which were applied in the TLC conditions.

The obtained results show that the chromatographic parameters determined by TLC under the tested conditions can be successfully used to describe the lipophilicity and evaluate the properties of structurally similar bioactive molecules. The presented indexes can also be involved in structure–retention relationship estimations, which allow one to create more effective target therapies for specific clinical applications.

## Figures and Tables

**Table 1:** Comparison of the value of the R_M0_ parameter for the investigated drugs with the use of different stationary phases in the methanol/water and acetone/water system.

Compound	RP-18	RP-8	RP-2	CN	DIOL	NH_2_
**methanol/water**						
P	1.7048	2.1593	0.4712	1.0852	0.1564	−0.1828
M	1.4589	1.8891	0.4114	0.6562	0.0194	−0.1844
T	1.1124	1.9067	0.2685	0.8520	0.3358	−0.2693
I	1.1091	1.7309	0.2371	0.7763	0.1040	−0.1833
C	4.4357	3.5729	2.6661	3.1469	0.0391	1.0244
E	3.9062	3.2291	1.8448	2.3315	1.3283	−0.2205
R	3.2075	2.2447	1.7526	1.5973	-0.0476	−0.1692
V	3.5034	2.6716	2.2324	2.4693	0.0301	−0.2128
CI	2.9465	2.8658	2.3930	2.6595	0.2266	−0.0704
F	3.1951	3.6857	2.3601	2.3322	0.2161	−0.2016
RB	3.5298	2.6288	1.1382	1.7518	0.4835	0.3421
**acetone/water**						
P	0.7572	1.3335	−0.0885	0.7418	0.1111	−0.6842
M	0.6441	1.1021	0.0757	0.7942	−0.3837	−0.5738
T	0.3949	0.8081	−0.1169	0.5707	−0.2370	−0.6496
I	0.4835	0.9569	−0.2855	0.7894	0.7019	−0.7145
C	3.7555	3.2575	2.3937	2.4404	0.3972	1.4811
E	2.5362	2.3901	1.7092	1.8886	0.9818	−0.5460
R	2.2732	2.0335	1.8214	1.5941	−0.0734	−0.3690
V	2.6983	2.3689	2.1503	1.8632	−0.0599	0.1163
CI	3.0736	3.0720	2.1178	1.9163	−0.1515	−0.6185
F	2.5679	2.7347	2.4046	1.9880	−0.4958	−0.6517
RB	2.8466	2.1131	2.9562	1.5364	0.0210	0.8523

**Table 2:** The R_M0_ values for the tested compounds obtained experimentally and with the use of calculation methods.

Method	P	M	T	I	C	E	R	V	CI	F	RB
**methanol/water**											
RP-18	1.70	1.46	1.11	1.11	4.44	3.91	3.21	3.50	2.95	3.20	3.53
RP-8	2.16	1.89	1.91	1.73	3.57	3.23	2.24	2.67	2.87	3.69	2.63
RP-2	0.47	0.41	0.27	0.23	2.67	1.84	1.75	2.23	2.39	2.36	1.14
CN	1.09	0.66	0.85	0.78	3.15	2.33	1.60	2.47	2.66	2.33	1.75
DIOL	0.16	0.02	0.33	0.10	0.04	1.33	−0.05	0.03	2.23	0.22	0.48
NH_2_	−0.18	−0.18	−0.27	−0.18	1.02	−0.22	−0.17	−0.21	−0.07	−0.20	0.34
AlogPs	2.20	2.28	2.42	2.61	3.99	3.70	3.32	2.32	3.21	2.98	4.68
ChemAxon	0.60	1.60	1.22	1.77	4.01	2.79	2.82	2.56	2.12	1.96	4.58
XlogP3	3.06	3.01	1.14	2.45	3.40	3.34	2.27	2.62	2.93	2.15	4.13
AC_logP	1.70	2.28	1.55	1.40	2.63	3.35	1.43	2.63	2.32	1.52	4.09
ALOGP	1.00	0.75	0.95	0.75	4.55	3.47	2.87	2.72	3.20	2.30	4.78
MLOGP	0.94	0.76	0.50	0.82	3.18	2.93	2.81	2.05	2.18	2.25	4.47
XLOGP2	2.65	1.60	-	2.98	3.85	3.94	3.22	3.15	3.27	2.97	4.21
**acetone/water**											
RP-18	0.76	0.64	0.39	0.48	3.76	2.54	2.27	2.70	3.07	2.57	2.85
RP-8	1.33	1.10	0.81	0.96	3.26	2.39	2.03	2.37	3.07	2.73	2.11
RP-2	−0.09	0.08	−0.12	−0.29	2.39	1.71	1.82	2.15	2.12	2.40	2.96
CN	0.74	0.79	0.57	0.79	2.44	1.89	1.59	1.86	1.92	1.99	1.54
DIOL	0.11	−0.38	−0.24	0.70	0.40	0.98	−0.07	−0.06	−0.15	−0.50	0.02
NH_2_	−0.68	−0.57	−0.65	−0.71	1.48	−0.55	−0.37	0.12	−0.62	−0.65	0.85
AlogPs	2.20	2.28	2.42	2.61	3.99	3.70	3.32	2.32	3.21	2.98	4.68
ChemAxon	0.60	1.60	1.22	1.77	4.01	2.79	2.82	2.56	2.12	1.96	4.58
XlogP3	3.06	3.01	1.14	2.45	3.40	3.34	2.27	2.62	2.93	2.15	4.13
AC_logP	1.70	2.28	1.55	1.40	2.63	3.35	1.43	2.63	2.32	1.52	4.09
ALOGP	1.00	0.75	0.95	0.75	4.55	3.47	2.87	2.72	3.20	2.30	4.78
MLOGP	0.94	0.76	0.50	0.82	3.18	2.93	2.81	2.05	2.18	2.25	4.47
XLOGP2	2.65	1.60	-	2.98	3.85	3.94	3.22	3.15	3.27	2.97	4.21

**Table 3:** The correlation coefficients (r) obtained for the linear correlation between experimental R_M0_ values calculated according to equations: R_M0(1)_ = b + a R_M0(2)_ in methanol/water system (white area) and in acetone/water system (grey area); R_M0_ = b + a logP_calc_ in methanol/water system (white area) and in acetone/water system (grey area); as well as logP_calc(1)_ = b + a logP_calc(2) (italic)_, respectively.

	RP-18	RP-8	RP-2	CN	DIOL	NH2	AlogPs	ChemAxon	XlogP3	AC_logP	ALOGP	MLOGP	XLOGP2
RP-18	-	0.9631	0.9434	0.9689	0.0818	0.6500	0.7089	0.7665	0.4854	0.4854	0.9213	0.8382	0.7306
RP-8	0.8375	-	0.8703	0.9726	0.0394	0.5023	0.5714	0.5869	0.3986	0.3784	0.8032	0.6948	0.5942
RP-2	0.8794	0.8699	-	0.8893	0.0746	0.5980	0.7392	0.7973	0.4559	0.5762	0.9077	0.8984	0.7086
CN	0.8997	0.8994	0.9630	-	0.1266	0.5561	0.6043	0.6724	0.3788	0.4065	0.8280	0.7388	0.6479
DIOL	0.2770	0.3095	0.0556	0.1695	-	0.1447	0.3048	0.2402	0.3588	0.2904	0.2141	0.1863	0.4908
NH2	0.5601	0.4639	0.4055	0.5266	0.1358	-	0.6676	0.8358	0.5566	0.5857	0.7615	0.6817	0.5766
AlogPs	0.7169	0.5315	0.4441	0.5232	0.3934	0.6523	-	*0.9003*	*0.6258*	*0.6955*	*0.8989*	*0.9251*	*0.8431*
ChemAxon	0.7841	0.4891	0.5198	0.5761	0.1759	0.7094	*0.9003*	-	*0.6123*	*0.7264*	*0.9136*	*0.9183*	*0.7771*
XlogP3	0.5156	0.3085	0.2234	0.5761	0.2741	0.5335	*0.6258*	*0.6123*	*-*	*0.7958*	*0.6207*	*0.6492*	*0.4777*
AC_logP	0.4854	0.3533	0.2585	0.3990	0.5422	0.4203	*0.6955*	*0.7264*	*0.7958*	*-*	*0.7265*	*0.7208*	*0.5940*
ALOGP	0.5848	0.6952	0.7237	0.7930	0.2895	0.6763	*0.8989*	*0.9136*	*0.6207*	*0.7265*	*-*	*0.9562*	*0.8678*
MLOGP	0.8658	0.6201	0.6163	0.6518	0.3106	0.5629	*0.9251*	*0.9183*	*0.6492*	*0.7208*	*0.9562*	*-*	*0.8583*
XLOGP2	0.7635	0.5438	0.5022	0.6344	0.4998	0.4966	*0.8431*	*0.7771*	*0.4777*	*0.5940*	*0.8678*	*0.8583*	*-*

## Data Availability

Data is contained within the article and supplementary material.

## Supplementary Materials

The following are available online at https://www.mdpi.com/1999-4923/13/4/440/s1, Table S1. Molecular information about analyzed substances. Table S2. The R_M_ values estimated by different TLC plates using methanol/water mobile phases. Table S3. The R_M_ values estimated by different TLC plates using acetone/water mobile phases. Table S4. Dependence of R_M_ value on the percentage of organic modifier (C) in the methanol/water phase system, according to the equation R_M_ = b + a%C. Table S5. Dependence of R_M_ value on the percentage of organic modifier (C) in the acetone/water phase system, according to the equation R_M_ = b + a%C. Table S6. Terms of the linear correlation according to equations RM_0(1)_ = b + a RM_0(2)_. Table S7. Correlation between logP_calc_ and chromatographic R_M0_ values according to the calibration equations: R_M0_ = b + alogP_calc_. Table S8. Terms of the linear correlation according to equations logP_calc(1)_ = b + a logP_calc(2)_.

## Abbreviations

WDI, World Drug Index; D, distribution coefficient; QSAR, quantitative structure–activity relationships; TLC, thin-layer chromatography; HPLC, high performance liquid chromatography; RP, reversed phase; NSAIDs, non-steroidal anti-inflammatory drugs; COX, cyclooxygenase; IAM, immobilized artificial membrane; PCA, Principal Component Analysis; CA, cluster analysis; Met, methanol; Ac, acetone; P, piroxicam; T, tenoxicam; M, meloxicam; I, isoxicam; C, celecoxib; E, etoricoxib; R, rofecoxib; V, valdecoxib; F, firocoxib; CI, cimicoxib; RB, robenacoxib.
