# Supplementary material for: Assessment of Lipophilicity Descriptors of Selected NSAIDs Obtained at Different TLC Stationary Phases
Source: Pharmaceutics. 2021 Mar 24;13(4):440. doi: 10.3390/pharmaceutics13040440 (PMC8064060; doi:10.3390/pharmaceutics13040440)
Supplement: Supplementary file 1 [file pharmaceutics-13-00440-s001.pdf]

pharmaceutics

MDPI

Supplementary Materials: Assessment of Lipophilicity Descriptors of Selected NSAIDs Obtained at Different TLC Stationary Phases

Małgorzata Starek, Alina Plenis, Marta Zagrobelna and Monika Dąbrowska

Table S1. Molecular information about analyzed substances.

Compound	Structural formula	Molecular formula	Molecular weight
Piroxicam	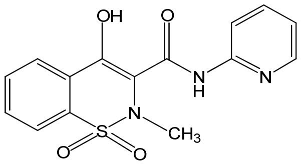	$C_{15}H_{13}N_3O_4S$	331.346
Meloxicam	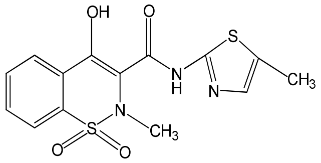	$C_{14}H_{13}N_3O_4S_2$	351.401
Tenoxicam	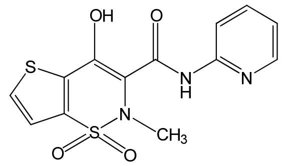	$C_{13}H_{11}N_3O_4S_2$	337.374
Isoxicam	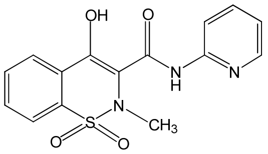	$C_{14}H_{13}N_3O_5S$	335.335
Celecoxib	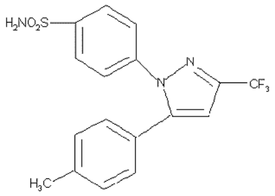	$C_{17}H_{14}F_3N_3O_2S$	314.359
Etoricoxib	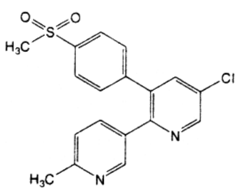	$C_{18}H_{15}ClN_2O_2S$	358.842
Rofecoxib	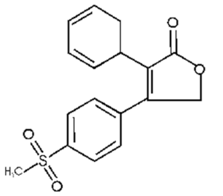	$C_{17}H_{14}O_4S$	314.356

Valdecoxib	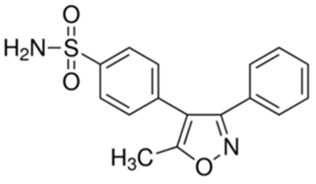	$C_{16}H_{14}N_2O_3S$	314.359
Cimicoxib	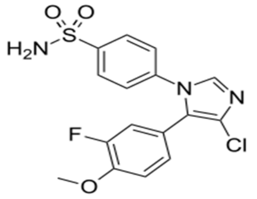	$C_{16}H_{13}ClFN_3O_3S$	381.809
Firocoxib	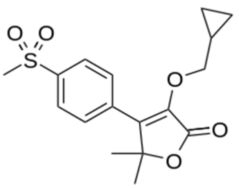	$C_{17}H_{20}O_5S$	336.4
Robenacoxib	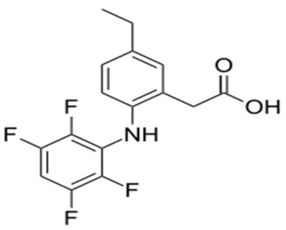	$C_{16}H_{13}F_4NO_2$	327.279

Table S2. The R_M values estimated by different TLC plates using methanol/water mobile phases.

	RP-18										
	**P**	**M**	**T**	**I**	**C**	**E**	**R**	**V**	**CI**	**F**	**RB**
0.30:0.70	0.95	0.79	0.50	0.50	-	-	1.99	-	-	-	-
0.35:0.65	0.72	0.55	0.33	0.33	-	-	1.69	1.99	1.69	-	-
0.40:0.60	0.58	0.39	0.21	0.21	-	1.99	1.28	1.51	1.28	1.70	1.99
0.45:0.55	0.25	0.16	0.02	0.03	-	1.69	1.12	1.28	1.06	1.19	1.69
0.50:0.50	0.18	0.03	−0.10	- 0.07	1.69	1.38	0.79	0.83	0.91	0.91	1.28
0.55:0.45	0.12	- 0.03	−0.19	- 0.18	1.38	1.06	0.55	0.72	0.63	0.72	1.19
0.60:0.40	- 0.07	- 0.10	−0.33	- 0.31	1.06	0.72	0.23	0.29	0.39	0.45	1.06
0.65:0.35	- 0.18	- 0.25	−0.37	- 0.35	0.79	0.52	0.07	0.12	0.23	0.33	0.83
0.70:0.30	- 0.35	- 0.41	−0.50	- 0.45	0.50	0.31	- 0.05	- 0.02	0.03	0.14	0.66
0.75:0.25	- 0.45	- 0.52	−0.60	- 0.60	0.25	0.18	- 0.18	- 0.16	- 0.07	- 0.07	0.39

0.80:0.20	- 0.52	- 0.60	−0.66	- 0.66	0.03	0.00	- 0.29	- 0.29	- 0.18	- 0.14	0.25
					**RP-8**						
0.30:0.70	1.38	0.95	1.06	1.00	-	-	-	-	-	-	-
0.35:0.65	1.06	0.87	0.91	0.91	-	-	-	-	-	-	-
0.40:0.60	0.95	0.83	0.83	0.87	-	1.70	0.95	1.19	1.19	-	-
0.45:0.55	0.72	0.50	0.45	0.50	-	1.19	0.69	0.89	1.06	-	1.06
0.50:0.50	0.58	0.37	0.27	0.37	1.51	1.06	0.52	0.66	0.87	1.38	0.87
0.55:0.45	0.41	0.21	0.16	0.25	0.69	0.63	0.29	0.39	0.66	1.00	0.63
0.60:0.40	0.33	0.09	0.02	0.18	0.58	0.45	0.12	0.18	0.58	0.69	0.41
0.65:0.35	0.12	- 0.07	−0.09	0.05	0.39	0.31	- 0.03	0.03	0.29	0.52	0.25
0.70:0.30	0,00	- 0.23	−0.25	- 0.10	0.29	0.16	- 0.19	- 0.14	0.03	0.31	0.12
0.75:0.25	- 0.12	- 0.33	−0.32	- 0.14	0.00	- 0.02	- 0.31	- 0.29	- 0.19	0.03	−0.07
0.80:0.20	- 0.18	- 0.45	−0.37	- 0.14	- 0.16	- 0.16	- 0.45	- 0.43	- 0.37	- 0.10	−0.18
					**RP-2**						
0.30:0.70	- 0.09	0.12	−0.09	- 0.07	-	-	-	-	1.51	1.51	0.60
0.35:0.65	- 0.19	- 0.02	−0.21	- 0.18	-	0.91	0.91	1.28	1.06	1.19	0.41
0.40:0.60	- 0.27	- 0.16	−0.35	- 0.27	-	0.63	0.63	0.87	0.83	0.83	0.25
0.45:0.55	- 0.35	- 0.29	−0.45	- 0.33	-	0.50	0.37	0.52	0.69	0.72	0.14
0.50:0.50	- 0.45	- 0.43	−0.52	- 0.60	1.06	0.45	0.19	0.35	0.45	0.52	−0.05
0.55:0.45	- 0.45	- 0.48	−0.60	- 0.66	0.48	0.14	- 0.07	0.03	0.27	0.41	−0.16
0.60:0.40	- 0.48	- 0.52	−0.66	- 0.69	0.29	- 0.09	- 0.27	- 0.17	0.10	0.27	−0.29
0.65:0.35	- 0.55	- 0.55	−0.69	- 0.69	0.25	- 0.10	- 0.29	- 0.19	- 0.09	0.12	−0.33
0.70:0.30	- 0.63	- 0.55	−0.69	- 0.69	0.23	- 0.14	- 0.29	- 0.19	- 0.12	- 0.03	−0.41
0.75:0.25	- 0.87	- 0.60	−0.81	- 0.72	- 0.03	- 0.25	- 0.41	- 0.37	- 0.37	- 0.23	−0.45
0.80:0.20	- 1.19	- 0.63	−0.83	- 0.72	- 0.29	- 0.37	- 0.48	- 0.45	- 0.48	- 0.37	−0.50
					**CN**						

0.30:0.70	0.55	0.43	0.35	0.35	-	-	-	-	1.70	1.51	1.06
0.35:0.65	0.33	0.16	0.27	0.21	-	1.28	0.69	1.38	1.38	1.19	0.72
0.40:0.60	0.14	-0.10	0.19	0.46	-	0.95	0.60	0.95	0.91	0.83	0.45
0.45:0.55	0.03	-0.27	−0.09	-0.16	1.19	0.69	0.55	0.69	0.83	0.72	0.31
0.50:0.50	-0.12	-0.43	−0.39	-0.39	0.79	0.50	0.53	0.52	0.66	0.60	0.02
0.55:0.45	-0.12	-0.43	−0.41	-0.39	0.55	0.35	0.33	0.31	0.45	0.41	−0.05
0.60:0.40	-0.12	-0.45	−0.43	-0.41	0.39	0.58	0.16	0.12	0.25	0.16	−0.14
0.65:0.35	-0.27	-0.50	−0.48	-0.41	0.09	0.03	0.03	-0.05	0.12	0.09	−0.23
0.70:0.30	-0.41	-0.52	−0.50	-0.41	-0.17	-0.16	-0.10	-0.21	-0.03	0.03	−0.37
0.75:0.25	-0.55	-0.55	−0.55	-0.43	-0.33	-0.23	-0.25	-0.33	-0.23	-0.17	−0.43
0.80:0.20	-0.69	-0.55	−0.60	-0.43	-0.43	-0.35	-0.29	-0.37	-0.37	-0.33	−0.66
**DIOL**											
0.30:0.70	0.00	-0.29	−0.09	-0.14	-0.23	0.79	-0.29	-0.31	-0.09	-0.48	0.03
0.35:0.65	-0.05	-0.37	−0.16	-0.27	-0.27	0.63	-0.33	-0.35	-0.09	-0.52	−0.05
0.40:0.60	-0.12	-0.45	−0.23	-0.41	-0.33	0.50	-0.39	-0.41	-0.12	-0.66	−0.12
0.45:0.55	-0.21	-0.55	−0.31	-0.48	-0.50	0.27	-0.50	-0.52	-0.21	-0.69	−0.27
0.50:0.50	-0.33	-0.69	−0.39	-0.55	-0.69	0.10	-0.63	-0.69	-0.29	-0.75	−0.37
0.55:0.45	-0.35	-0.69	−0.39	-0.39	-0.69	0.07	-0.63	-0.69	-0.33	-0.83	−0.43
0.60:0.40	-0.37	-0.69	−0.41	-0.69	-0.72	0.03	-0.66	-0.72	-0.37	-0.87	−0.48
0.65:0.35	-0.39	-0.75	−0.50	-0.69	-0.74	-0.05	-0.69	-0.79	-0.39	-0.91	−0.55
0.70:0.30	-0.41	-0.83	−0.69	-0.72	-0.72	-0.12	-0.72	-0.83	-0.41	-0.91	−0.63
0.75:0.25	-0.41	-0.87	−0.75	-0.72	-0.75	-0.23	-0.72	-0.87	-0.52	-0.95	−0.72

0.80:0.20	-	-	−0.79	-	-	-	-	-	-	-	−0.75
	0.43	0.91		0.79	0.75	0.37	0.75	0.87	0.55	1.00	
					**NH_2**						
0.30:0.70	-	-	−0.43	-	0.07	-	-	-	-	-	−0.05
	0.39	0.41		0.48		0.63	0.52	0.50	0.35	0.50	
0.35:0.65	-	-	−0.43	-	0.05	-	-	-	-	-	−0.09
	0.43	0.45		0.50		0.69	0.55	0.55	0.39	0.50	
0.40:0.60	-	-	−0.45	-	0.03	-	-	-	-	-	−0.14
	0.48	0.50		0.52		0.72	0.55	0.55	0.41	0.55	
0.45:0.55	-	-	−0.52	-	0.00	-	-	-	-	-	−0.18
	0.50	0.52		0.58		0.72	0.69	0.60	0.48	0.63	
0.50:0.50	-	-	−0.60	-	-	-	-	-	-	-	−0.23
	0.55	0.55		0.63	0.02	0.72	0.75	0.63	0.50	0.75	
0.55:0.45	-	-	−0.60	-	-	-	-	-	-	-	−0.23
	0.60	0.58		0.63	0.35	0.75	0.79	0.83	0.63	0.75	
0.60:0.40	-	-	−0.60	-	-	-	-	-	-	-	−0.23
	0.63	0.63		0.63	0.79	0.79	0.87	0.83	0.66	0.79	
0.65:0.35	-	-	−0.63	-	-	-	-	-	-	-	−0.35
	0.66	0.66		0.69	0.83	0.95	0.95	0.87	0.69	0.79	
0.70:0.30	-	-	−0.63	-	-	-	-	-	-	-	−0.41
	0.69	0.72		0.75	0.87	1.06	1.00	0.87	0.69	0.79	
0.75:0.25	-	-	−0.66	-	-	-	-	-	-	-	−0.58
	0.72	0.75		0.87	0.95	1.19	1.00	0.91	0.75	0.91	
0.80:0.20	-	-	−0.69	-	-	-	-	-	-	-	−0.69
	0.75	0.79		0.95	1.00	1.19	1.00	0.91	0.79	1.00	

Table S3. The R_M values estimated by different TLC plates using acetone/water mobile phases.

					**RP-18**						
	**P**	**M**	**T**	**I**	**C**	**E**	**R**	**V**	**CI**	**F**	**RB**
0.30:0.70	0.31	0.10	−0.10	0.03	-	1.69	1.38	1.69	1.99	1.69	1.99
0.35:0.65	0.19	0.02	−0.16	-	-	1.38	1.28	1.51	1.70	1.51	1.69
				0.03							
0.40:0.60	0.12	-	−0.21	-	-	1.12	0.95	1.19	1.28	1.06	1.38
		0.05		0.07							
0.45:0.55	0.05	-	−0.25	-	1.69	0.83	0.66	0.87	1.06	0.87	1.06
		0.12		0.09							
0.50:0.50	0.03	-	−0.33	-	1.38	0.60	0.43	0.60	0.83	0.72	0.87
		0.16		0.18							
0.55:0.45	0.02	-	−0.37	-	1.06	0.50	0.33	0.48	0.72	0.58	0.72
		0.23		0.19							
0.60:0.40	-	-	−0.41	-	0.83	0.35	0.21	0.33	0.39	0.45	0.69
	0.03	0.29		0.23							

0.65:0.35	-0.18	-0.41	−0.55	-0.37	0.50	0.16	0.02	0.12	0.21	0.33	0.45
0.70:0.30	-0.31	-0.52	−0.60	-0.45	0.37	0.12	-0.03	0.09	0.07	0.19	0.33
0.75:0.25	-0.33	-0.60	−0.72	-0.50	0.19	-0.03	-0.16	-0.10	-0.02	-0.03	0.21
0.80:0.20	-0.55	-0.83	−0.91	-0.72	0.00	-0.14	-0.25	-0.21	-0.16	-0.10	0.12
**RP-8**											
0.30:0.70	0.72	0.52	0.31	0.50	-	1.39	1.28	1.38	-	-	-
0.35:0.65	0.58	0.39	0.15	0.35	-	1.28	1.00	1.28	1.70	-	-
0.40:0.60	0.45	0.27	0.03	0.27	-	1.19	0.91	1.19	1.38	1.38	0.95
0.45:0.55	0.33	0.16	−0.03	0.21	-	0.95	0.63	0.76	1.19	1.19	0.83
0.50:0.50	0.21	0.03	−0.10	0.16	1.51	0.69	0.55	0.66	0.83	0.91	0.66
0.55:0.45	0.12	-0.05	−0.18	0.07	0.83	0.43	0.33	0.39	0.72	0.83	0.50
0.60:0.40	0.03	-0.18	−0.25	0.02	0.63	0.29	0.18	0.23	0.52	0.72	0.27
0.65:0.35	-0.07	-0.29	−0.35	-0.07	0.45	0.14	0.03	0.09	0.23	0.50	0.18
0.70:0.30	-0.18	-0.37	−0.45	-0.18	0.33	0.05	-0.07	-0.02	0.05	0.39	0.03
0.75:0.25	-0.31	-0.41	−0.58	-0.29	0.18	-0.05	-0.12	-0.09	-0.10	0.12	−0.10
0.80:0.20	-0.39	-0.52	−0.66	-0.37	0.12	-0.10	-0.16	-0.14	-0.18	-0.05	−0.16
**RP-2**											
0.30:0.70	-0.21	-0.10	−0.17	-0.43	-	0.91	1.06	-	1.19	-	-
0.35:0.65	-0.45	-0.31	−0.41	-0.52	-	0.79	0.83	1.19	1.00	-	-
0.40:0.60	-0.66	-0.45	−0.66	-0.66	-	0.69	0.66	0.87	0.91	-	1.38
0.45:0.55	-0.75	-0.45	−0.72	-0.79	1.06	0.45	0.43	0.58	0.79	1.00	1.06
0.50:0.50	-0.83	-0.48	−0.72	-0.79	0.91	0.31	0.29	0.43	0.58	0.79	0.79
0.55:0.45	-0.83	-0.58	−0.75	-0.83	0.31	0.05	0.03	0.09	0.43	0.69	0.58
0.60:0.40	-0.83	-0.66	−0.75	-0.83	0.05	-0.10	-0.14	-0.14	0.27	0.52	0.43

0.65:0.35	-	-	−0.75	-	0.00	-	-	-	0.12	0.29	0.18
	0.87	0.66		0.87		0.21	0.21	0.19			
0.70:0.30	-	-	−0.75	-	-	-	-	-	0.02	0.16	−0.14
	0.87	0.69		0.87	0.03	0.27	0.29	0.25			
0.75:0.25	-	-	−0.83	-	-	-	-	-	-	0.03	−0.21
	0.95	0.69		0.91	0.09	0.31	0.33	0.29	0.21		
0.80:0.20	-	-	−0.87	-	-	-	-	-	-	-	−0.27
	0.95	0.75		0.91	0.12	0.35	0.39	0.33	0.35	0.10	
**CN**											
0.30:0.70	0.27	0.10	0.00	0.14	-	0.91	0.79	0.87	0.95	1.06	0.79
0.35:0.65	0.16	-	−0.05	0.00	1.06	0.87	0.63	0.69	0.83	0.91	0.58
		0.05									
0.40:0.60	0.07	-	−0.12	-	0.95	0.79	0.50	0.55	0.58	0.87	0.28
		0.23		0.13							
0.45:0.55	-	-	−0.29	-	0.79	0.50	0.37	0.43	0.55	0.69	0.23
	0.07	0.35		0.23							
0.50:0.50	-	-	−0.45	-	0.66	0.29	0.23	0.35	0.43	0.45	0.05
	0.19	0.48		0.35							
0.55:0.45	-	-	−0.55	-	0.31	0.09	0.05	0.09	0.09	0.37	−0.03
	0.29	0.55		0.43							
0.60:0.40	-	-	−0.69	-	0.05	-	-	-	-	0.23	−0.18
	0.39	0.66		0.52		0.10	0.14	0.14	0.14		
0.65:0.35	-	-	−0.72	-	-	-	-	-	-	0.05	−0.35
	0.45	0.72		0.60	0.14	0.23	0.29	0.33	0.29		
0.70:0.30	-	-	−0.75	-	-	-	-	-	-	-	−0.45
	0.52	0.75		0.91	0.35	0.37	0.43	0.52	0.41	0.14	
0.75:0.25	-	-	−0.87	-	-	-	-	-	-	-	−0.60
	0.52	0.79		0.91	0.41	0.41	0.45	0.58	0.45	0.29	
0.80:0.20	-	-	−0.87	-	-	-	-	-	-	-	−0.69
	0.58	0.79		0.95	0.45	0.43	0.50	0.66	0.52	0.35	
**DIOL**											
0.30:0.70	-	-	−0.41	0.23	-	0.53	-	-	-	-	−0.31
	0.31	0.69			0.29		0.48	0.48	0.39	0.72	
0.35:0.65	-	-	−0.52	-	-	0.29	-	-	-	-	−0.39
	0.43	0.69		0.10	0.39		0.63	0.63	0.48	0.72	
0.40:0.60	-	-	−0.66	-	-	0.09	-	-	-	-	−0.48
	0.55	0.69		0.52	0.53		0.79	0.79	0.52	0.75	
0.45:0.55	-	-	−0.66	-	-	-	-	-	-	-	−0.53
	0.58	0.72		0.69	0.69	0.02	0.79	0.75	0.55	0.75	
0.50:0.50	-	-	−0.69	-	-	-	-	-	-	-	−0.63
	0.63	0.75		0.87	0.87	0.09	0.83	0.87	0.58	0.83	

0.55:0.45	-	-	−0.72	-	-	-	-	-	-	-	−0.69
	0.66	0.83		0.95	0.95	0.16	0.91	0.95	0.63	0.83	
0.60:0.40	-	-	−0.75	-	-	-	-	-	-	-	−0.75
	0.69	0.95		1.00	1.06	0.21	1.00	1.00	0.66	0.87	
0.65:0.35	-	-	−0.79	-	-	-	-	-	-	-	−0.83
	0.85	1.00		1.12	1.19	0.33	1.12	1.12	0.72	0.95	
0.70:0.30	-	-	−0.83	-	-	-	-	-	-	-	−0.87
	1.00	1.12		1.19	1.28	0.45	1.19	1.19	0.79	0.95	
0.75:0.25	-	-	−0.87	-	-	-	-	-	-	-	−0.87
	1.06	1.12		1.19	1.28	0.37	1.28	1.19	0.83	1.00	
0.80:0.20	-	-	−0.91	-	-	-	-	-	-	-	−0.91
	1.06	1.19		1.28	1.51	0.69	1.28	1.38	0.83	1.00	
**NH_2**											
0.30:0.70	-	-	−0.69	-	0.14	-	-	-	-	-	0.05
	0.75	0.69		0.83		0.87	0.87	0.69	0.72	0.79	
0.35:0.65	-	-	−0.75	-	0.05	-	-	-	-	-	−0.12
	0.79	0.75		0.83		0.91	0.91	0.72	0.79	0.82	
0.40:0.60	-	-	−0.79	-	-	-	-	-	-	-	−0.29
	0.83	0.79		0.87	0.03	1.19	1.19	0.72	0.79	0.83	
0.45:0.55	-	-	−0.79	-	-	-	-	-	-	-	−0.37
	0.83	0.79		0.87	0.27	1.19	1.19	0.87	0.82	0.83	
0.50:0.50	-	-	−0.79	-	-	-	-	-	-	-	−0.63
	0.83	0.79		0.87	0.52	1.19	1.19	0.87	0.83	0.87	
0.55:0.45	-	-	−0.79	-	-	-	-	-	-	-	−0.72
	0.83	0.83		0.95	0.79	1.19	1.19	1.12	0.87	0.87	
0.60:0.40	-	-	−0.79	-	-	-	-	-	-	-	−0.87
	0.87	0.83		0.95	1.19	1.19	1.19	1.19	0.87	0.91	
0.65:0.35	-	-	−0.79	-	-	-	-	-	-	-	−0.91
	0.87	0.91		0.95	1.38	1.38	1.38	1.28	0.91	0.95	
0.70:0.30	-	-	−0.79	-	-	-	-	-	-	-	−1.19
	0.91	0.91		0.95	1.51	1.51	1.51	1.51	0.91	0.95	
0.75:0.25	-	-	−0.87	-	-	-	-	-	-	-	−1.19
	0.91	0.91		1.00	1.51	1.51	1.69	1.69	0.95	1.00	
0.80:0.20	-	-	−0.87	-	-	-	-	-	-	-	−1.38
	0.91	0.95		1.00	1.69	1.51	1.69	1.69	0.95	1.00	

Table S4. Dependence of R_M value on the percentage of organic modifier (C) in the methanol/water phase system, according to the equation $R_M = b + a\%C$.

	a	S_a	b(~R_{M0})	S_b	r	r^2	S_e
			**RP−18**				
P	−2.8964	0.1488	1.7048	0.0851	0.9883	0.9768	0.0780
M	−2.6509	0.1342	1.4589	0.0768	0.9886	0.9774	0.0703
T	−2.3018	0.0989	1.1124	0.0566	0.9918	0.9837	0.0519
I	−2.2727	0.0877	1.1091	0.0502	0.9937	0.9868	0.0460
C	−5.5714	0.1446	4.4357	0.0951	0.9983	0.9966	0.0382
E	−5.0567	0.2744	3.9062	0.1684	0.9898	0.9798	0.1063
R	−4.6418	0.2654	3.2075	0.1519	0.9856	0.9714	0.1392
V	−5.0024	0.3499	3.5034	0.2073	0.9810	0.9623	0.1589
CI	−4.0860	0.2184	2.9465	0.1294	0.9888	0.9777	0.0992
F	−4.3567	0.3406	3.1951	0.2090	0.9793	0.9590	0.1319
RB	−4.1533	0.2247	3.5298	0.1379	0.9899	0.9799	0.0870
			**RP−8**				
P	−3.0582	0.1362	2.1593	0.0779	0.9911	0.9824	0.0714
M	−2.9818	0.1082	1.8891	0.0619	0.9941	0.9882	0.0567
T	−3.0255	0.1871	1.9067	0.1070	0.9832	0.9667	0.0981
I	−2.5273	0.1838	1.7309	0.1052	0.9770	0.9545	0.0963
C	−4.7714	0.7654	3.5729	0.5033	0.9412	0.8860	0.2024
E	−4.3967	0.3552	3.2291	0.2180	0.9779	0.9563	0.1375
R	−3.4467	0.1390	2.2447	0.0852	0.9943	0.9887	0.0538
V	−3.9933	0.2001	2.6716	0.1233	0.9912	0.9825	0.0778
CI	−4.0133	0.1528	2.8658	0.0938	0.9950	0.9899	0.0592
F	−4.8286	0.2942	2.6857	0.1935	0.9908	0.9818	0.0778
RB	−3.5881	0.1510	2.6288	0.0960	0.9947	0.9895	0.0489
			**RP−2**				
P	−1.7691	0.2171	0.4712	0.1242	0.9384	0.8805	0.1138
M	−1.4273	0.1755	0.4114	0.1004	0.9381	0.8801	0.0920
T	−1.4800	0.1228	0.2685	0.0702	0.9703	0.9416	0.0643
I	−1.3600	0.2009	0.2371	0.1149	0.8359	0.8176	0.1053
C	−3.6643	0.6162	2.6661	0.4052	0.9360	0.8761	0.1630
E	−3.8449	0.2595	1.8448	0.1537	0.9683	0.9376	0.1178
R	−2.9976	0.3311	1.7526	0.1962	0.9110	0.8999	0.1503
V	−3.5903	0.4183	2.2324	0.2479	0.9020	0.8897	0.1899
CI	−3.7146	0.2030	2.3930	0.1162	0.9868	0.9738	0.1065
F	−3.4746	0.1815	2.3601	0.1039	0.9879	0.9760	0.0962
RB	−2.2000	0.1610	1.1382	0.0921	0.9767	0.9540	0.0844
			**CN**				
P	−2.1764	0.1485	1.0852	0.0850	0.9796	0.9597	0.0778
M	−1.1724	0.3097	0.6562	0.1772	0.8803	0.7749	0.1623

T	−1.9855	0.2620	0.8520	0.1499	0.9298	0.8645	0.1374
I	−1.7436	0.3912	0.7763	0.2238	0.8296	0.6882	0.2051
C	−4.6191	0.2660	3.1469	0.1689	0.9902	0.9805	0.1689
E	−3.4739	0.1987	2.3315	0.1117	0.9872	0.9745	0.0902
R	−2.3867	0.1358	1.5973	0.0804	0.9873	0.9747	0.0616
V	−3.7709	0.2668	2.4693	0.1581	0.9805	0.9615	0.1581
CI	−3.8982	0.2161	2.6595	0.1237	0.9864	0.9731	0.1133
F	−3.4073	0.2032	2.3322	0.1163	0.9844	0.9690	0.1065
RB	−3.0727	0.2335	1.7518	0.1336	0.9750	0.9506	0.1224
**DIOL**							
P	−0.8061	0.1255	0.1564	0.0744	0.9151	0.8374	0.0570
M	−1.2073	0.0900	0.0194	0.0515	0.9758	0.9523	0.0515
T	−1.3891	0.0906	0.3358	0.0518	0.9813	0.9630	0.0518
I	−1.1891	0.1245	0.1040	0.0712	0.9540	0.9101	0.0652
C	−1.1273	0.1851	0.0391	0.1059	0.8970	0.8046	0.0970
E	−2.1473	0.1555	1.3283	0.0889	0.9772	0.9549	0.0889
R	−0.9564	0.1077	−0.0476	0.0616	0.9473	0.8974	0.0616
V	−1.2200	0.1040	0.0301	0.0595	0.9688	0.9385	0.0545
CI	−0.9691	0.0532	0.2266	0.0304	0.9867	0.9736	0.0279
F	−1.0236	0.0766	0.2161	0.0439	0.9757	0.9520	0.0402
RB	−1.5964	0.0638	0.4835	0.0365	0.9929	0.9858	0.0334
**NH_2**							
P	−0.7255	0.0205	−0.1828	0.1117	0.9964	0.9928	0.0107
M	−0.7491	0.0195	−0.1844	0.0111	0.9969	0.9939	0.0102
T	−0.5418	0.0557	−0.2693	0.0318	0.9555	0.9130	0.0292
I	−0.8618	0.0889	−0.1833	0.0508	0.9552	0.9125	0.0466
C	−2.6327	0.3004	1.0244	0.1719	0.9461	0.8951	0.1575
E	−1.1546	0.1514	−0.2205	0.0866	0.9306	0.8660	0.0793
R	−1.1255	0.0795	−0.1692	0.4554	0.9782	0.9570	0.0416
V	−0.9436	0.0978	−0.2128	0.5590	0.9548	0.9117	0.0513
CI	−0.9200	0.0550	−0.0704	0.0315	0.9843	0.9688	0.0289
F	−0.9491	0.0827	−0.2016	0.0473	0.9675	0.9360	0.0434
RB	−1.1509	0.1253	0.3421	0.0717	0.9506	0.9037	0.0657

a – slope, S_a – standard error of the slope, b – intercept, S_b – standard error of the intercept, r, r^2– correlation coefficients, S_e – standard error of the estimate.

Table S5. Dependence of R_M value on the percentage of organic modifier (C) in the acetone/water phase system, according to the equation $R_M = b + a\%C$.

	a	S_a	b(~R_{M0})	S_b	r	r^2	S_e
**RP−18**							
P	−1.4891	0.1261	0.7572	0.0721	0.9692	0.9393	0.0661
M	−1.6818	0.1127	0.6441	0.0645	0.9804	0.9611	0.0591
T	−1.4800	0.1114	−0.3941	0.0638	0.9754	0.9514	0.0584
I	−1.3418	0.1208	0.4835	0.0691	0.9654	0.9319	0.0633
C	−4.8048	0.2580	3.7555	0.1640	0.9915	0.9830	0.0836
E	−3.5236	0.2432	2.5362	0.1392	0.9792	0.9589	0.1275
R	−3.3364	0.2336	2.2732	0.1337	0.9786	0.9577	0.1224
V	−3.8200	0.2446	2.6983	0.0139	0.9820	0.9644	0.1283
CI	−4.2546	0.2447	3.0736	0.1401	0.9854	0.9711	0.1283
F	−3.4673	0.2193	2.5679	0.1255	0.9825	0.9652	0.1150
RB	−3.6036	0.2640	2.8466	0.1511	0.9767	0.9539	0.1384
**RP−8**							
P	−2.1782	0.0428	1.3335	0.0245	0.9982	0.9965	0.0224
M	−2.0782	0.0642	1.1021	0.0367	0.9957	0.9914	0.0336
T	−1.8182	0.0585	0.8081	0.0334	0.9954	0.9907	0.0306
I	−1.6291	0.0571	0.9569	0.0326	0.9945	0.9890	0.0299
C	−4.1214	0.7404	3.2575	0.4869	0.9279	0.8610	0.1959
E	−3.3109	0.1925	2.3901	0.1101	0.9851	0.9704	0.1009
R	−2.9436	0.1796	2.0335	0.1027	0.9836	0.9675	0.0941
V	−3.3600	0.2237	2.3689	0.1280	0.9806	0.9616	0.1173
CI	−4.2400	0.1884	3.0720	0.1117	0.9922	0.9845	0.0856
F	−3.4467	0.1403	2.7347	0.0861	0.9942	0.9885	0.0543
RB	−2.9367	0.1222	2.1131	0.0750	0.9940	0.9880	0.0473
**RP−2**							
P	−1.1946	0.2248	−0.0885	0.1286	0.8708	0.7583	0.1178
M	−1.0964	0.1391	0.0757	0.0795	0.9346	0.8735	0.0729
T	−1.0073	0.2334	−0.1169	0.1335	0.8211	0.6741	0.1224
I	−0.8709	0.1400	−0.2855	0.0801	0.9007	0.8113	0.0734
C	−3.4119	0.6962	2.3937	0.4424	0.8944	0.8001	0.2256
E	−2.7836	0.1958	1.7092	0.1120	0.9784	0.9574	0.1120
R	−2.9909	0.2244	1.8214	0.1284	0.9756	0.9518	0.1284
V	−2.3988	0.3868	2.1503	0.2292	0.9519	0.9061	0.1756
CI	−3.0655	0.0589	2.1178	0.0337	0.9983	0.9967	0.0309
F	−3.1714	0.1059	2.4046	0.0673	0.9967	0.9934	0.0343
RB	−4.2233	0.2439	2.9562	0.1497	0.9885	0.9772	0.0945
**CN**							
P	−1.7636	0.1141	0.7418	0.0652	0.9816	0.9637	0.0598
M	−2.1400	0.1603	0.7942	0.0917	0.9756	0.9519	0.0840

T	−1.9249	0.1551	0.5707	0.0919	0.9750	0.9506	0.0704
I	−2.2436	0.1059	0.7894	0.0605	0.9901	0.9803	0.0555
C	−3.8146	0.2282	2.4404	0.1352	0.9859	0.9721	0.1036
E	−3.1182	0.1996	1.8886	0.1142	0.9821	0.9644	0.1046
R	−2.7727	0.1234	1.5941	0.0706	0.9912	0.9825	0.0647
V	−3.2636	0.1297	1.8632	0.0742	0.9929	0.9859	0.0680
CI	−3.2164	0.1848	1.9163	0.1058	0.9855	0.9711	0.0969
F	−2.9782	0.0878	1.9880	0.0502	0.9961	0.9922	0.0460
RB	−2.8545	0.1197	1.5364	0.0685	0.9922	0.9844	0.0627
**DIOL**							
P	−1.4946	0.0998	0.1111	0.0570	0.9805	0.9614	0.0523
M	−0.9800	0.0822	−0.3837	0.0470	0.9697	0.9405	0.0430
T	−0.8600	0.0809	−0.2370	0.0463	0.9624	0.9261	0.0424
I	−2.7109	0.3615	0.7019	0.2069	0.9284	0.8620	0.2069
C	−2.3818	0.1012	0.3972	0.0578	0.9919	0.9840	0.0530
E	−2.0182	0.1739	0.9818	0.0995	0.9681	0.9373	0.0912
R	−1.5691	0.0857	−0.0734	0.0490	0.9868	0.9738	0.0449
V	−1.6018	0.0905	−0.0599	0.0518	0.9859	0.9720	0.0474
CI	−0.8782	0.0370	−0.1515	0.0212	0.9921	0.9843	0.0194
F	−0.6473	0.0438	−0.4958	0.0251	0.9800	0.9604	0.0230
RB	−1.2382	0.0713	0.0210	0.0408	0.9854	0.9710	0.0374
**NH_2**							
P	−0.2982	0.0306	−0.6842	0.0174	0.9558	0.9135	0.0160
M	−0.4691	0.0394	−0.5738	0.0225	0.9696	0.9401	0.0206
T	−0.2618	0.0424	−0.6496	0.0277	0.8744	0.7644	0.0253
I	−0.3655	0.0356	−0.7145	0.0203	0.9597	0.9211	0.0186
C	−4.1309	0.2491	1.4811	0.1425	0.9840	0.9683	0.1306
E	−1.2618	0.1541	−0.5460	0.0881	0.9389	0.8817	0.0807
R	−1.1620	0.1400	−0.3690	0.0841	0.9648	0.9309	0.0770
V	−2.2527	0.1641	0.1163	0.0939	0.9769	0.9544	0.0860
CI	−0.4309	0.0285	−0.6185	0.0163	0.9809	0.9621	0.0149
F	−0.4382	0.0271	−0.6517	0.0155	0.9832	0.9667	0.0142
RB	−2.8091	0.1029	0.8523	0.0589	0.9940	0.9881	0.0540

a – slope, S_a – standard error of the slope, b – intercept, S_b – standard error of the intercept, r, r^2– correlation coefficients, S_e – standard error of the estimate.

Table S6. Terms of the linear correlation according to equations $RM_{0(1)} = b + a\ RM_{0(2)}$.

Stationary phases (1)/(2)	a	S_a	b	S_b	r	r^2	S_e
			**methanol/water**				
RP-18/RP-8	1.4493	0.3152	−1.0288	0.8446	0.8375	0.7013	0.6808
RP-18/RP-2	1.0916	0.1970	−1.1741	0.3341	0.8794	0.7733	0.5931
RP-18/CN	1.2396	0.2004	0.5216	0.3945	0.8997	0.8095	0.5438
RP-18/DIOL	0.8479	0.9803	−2.5154	0.4434	0.2770	0.0767	1.1971
RP-18/NH_2	1.7191	0.8475	2.7881	0.3122	0.5601	0.3137	1.0322
RP-8/RP-2	0.6240	0.1179	1.7051	0.2000	0.8699	0.7567	0.3551
RP-8/CN	0.7113	0.1192	1.3272	0.2343	0.8994	0.7988	0.3234
RP-8/DIOL	0.547	0.5606	2.4552	0.2536	0.3095	0.0958	0.6845
RP-8/NH_2	0.8227	0.5237	2.6230	0.1929	0.4639	0.2152	0.6378
RP-2/CN	1.0688	0.0996	−0.4785	0.1959	0.9630	0.9274	0.2703
RP-2/DIOL	0.1371	0.8206	1.3967	0.3712	0.0556	0.0031	1.0021
RP-2/NH_2	1.0025	0.7533	1.4619	0.2775	0.4055	0.1644	0.9175
CN/DIOL	0.3766	0.7298	1.6892	0.3301	0.1695	0.0287	0.8912
CN/NH_2	1.1731	0.6313	1.8123	0.2385	0.5266	0.2778	0.7688
DIOL/NH_2	−0.1361	0.3311	0.2588	0.1219	0.1358	0.0184	0.4033
			**acetone/water**				
RP-18/RP-8	1.3523	0.1259	−0.7215	0.2738	0.9631	0.9275	0.3408
RP-18/RP-2	0.9318	0.1060	0.7211	0.1909	0.9434	0.8957	0.4091
RP-18/CN	1.8340	0.1559	−0.6850	0.2472	0.9689	0.9389	0.3129
RP-18/DIOL	0.2171	0.8823	1.9867	0.3861	0.0818	0.0067	1.2622
RP-18/NH_2	1.0642	0.4147	2.2301	0.3034	0.6500	0.4225	0.9624
RP-8/RP-2	0.0610	0.1151	1.1751	0.2074	0.8703	0.7574	0.4442
RP-8/CN	1.3111	0.1044	0.0931	0.1655	0.9726	0.9460	0.2095
RP-8/DIOL	0.0746	0.6300	2.0090	0.2757	0.0394	0.0015	0.9012
RP-8/NH_2	0.5856	0.3361	−1.1298	0.4646	0.8893	0.7908	0.5582
RP-2/CN	1.7095	0.2931	−1.1298	0.5656	0.8893	0.7908	0.5882
RP-2/DIOL	−0.2013	0.8966	1.3903	0.3923	0.0746	0.0055	1.2826
RP-2/NH_2	0.9943	0.4442	1.5878	0.3250	0.5980	0.3576	1.0309
CN/DIOL	0.1776	0.4640	1.4524	0.2030	0.1266	0.0160	0.6637
CN/NH2	0.4810	0.2396	1.5682	0.1753	0.5561	0.3093	0.5561
DIOL/NH_2	0.0892	0.0203	0.0927	0.1487	0.1447	0.0209	0.4718

a – slope, S_a – standard error of the slope, b – intercept, S_b – standard error of the intercept, r, r^2– correlation coefficients, S_e – standard error of the estimate.

Table S7. Correlation between $logP_{calc}$ and chromatographic R_{M0} values according to the calibration equations: R_{M0} = b + $alogP_{calc}$.

Stationary phase	Organic modifier	a	S_a	b	S_b	r	r^2	S_e
**AlogPs**								
RP-18	M	1.0508	0.3406	−0.4823	1.0761	0.7169	0.5140	0.8685
	A	1.0561	0.3503	−1.2339	1.1067	0.7089	0.5025	0.8932
RP-8	M	0.4501	0.2391	1.2196	0.7556	0.5315	0.2824	0.6098
	A	0.6063	0.2903	0.1564	0.9171	0.5714	0.3265	0.7402
RP-2	M	0.5244	0.3526	−0.1743	1.1142	0.4441	0.1972	0.8992
	A	1.1186	0.3397	−2.0525	1.0732	0.7392	0.5465	0.8662
CN	M	0.5567	0.3022	0.0822	0.9548	0.5232	0.2738	0.7707
	A	0.4757	0.2090	0.0077	0.6605	0.6043	0.3652	0.5331
DIOL	M	0.1884	0.1467	−0.3146	0.4637	0.3934	0.1548	0.3742
	A	0.1710	0.1781	−0.4504	0.5627	0.3048	0.0929	0.4541
NH_2	M	0.3115	0.1207	−0.9837	0.3812	0.6523	0.4255	0.3077
	A	0.6075	0.2258	−2.0756	0.7136	0.6676	0.4457	0.5759
**ChemAxon**								
RP-18	M	0.7939	0.2094	0.8595	0.5478	0.7841	0.6148	0.7733
	A	0.7889	0.2203	0.1359	0.5761	0.7665	0.5875	0.8132
RP-8	M	0.2861	0.1701	1,9219	0.4448	0.4891	0.2392	0.6279
	A	0.4302	0.1978	0.9966	0.5173	0.5869	0.3444	0.7303
RP-2	M	0.4240	0.2323	0.4293	0.6073	0.5198	0.2702	0.1892
	A	0.8334	0.2103	−0.5968	0.5499	0.7973	0.6357	0.7763
CN	M	0.4233	0.2002	0.7863	0.5236	0.5761	0.3312	0.7392
	A	0.3656	0.1341	0.6005	0.3508	0.6724	0.4522	0.4952
DIOL	M	0.0582	0.1085	0.1250	0.2838	0.1759	0.0309	0.4007
	A	0.0931	0.1254	−0.1466	0.3279	0.2402	0.0578	0.4628
NH_2	M	0.2340	0.0775	−0.5829	0.2027	0.7094	0.5033	0.4481
	A	0.5254	0.1150	−1.4569	0.3009	0.8358	0.6950	0.4247
**XlogP3**								
RP-18	M	0.7768	0.4303	0.5841	1.2357	0.5156	0.2658	1.0675
	A	0.7433	0.4463	−0.0583	1.2817	0.4854	0.2356	1.0673
RP-8	M	0.2686	0.9729	1.8544	0.7927	0.3085	0.0952	0.6848
	A	0.4347	0.3334	0.8092	0.9576	0.3986	0.1588	0.8272
RP-2	M	0.2712	0.3943	0.6808	1.1324	0.2234	0.0499	0.9782
	A	0.7092	0.4614	−0.5909	1.3251	0.4559	0.2079	1.1447
CN	M	0.4234	0.2002	0.7863	0.5236	0.5761	0.3319	0.7392
	A	0.3065	0.2496	0.6155	0.7168	0.3788	0.1435	0.6192
DIOL	M	0.1349	0.1578	−0.1114	0.4531	0.2741	0.0751	0.3915
	A	0.2069	0.1794	−0.5001	0.5152	0.3588	0.1287	0.4451
NH_2	M	0.2619	0.1384	−0.7553	0.3974	0.5335	0.2847	0.3433

	A	0.5207	0.2590	−1.6574	0.7439	0.5566	0.3098	0.6426
				**AC_logP**				
RP-18	M	0.7768	0.4303	0.5841	1.2357	0.5156	0.2658	1.0673
	A	0.7433	0.4463	−0.0583	1.2817	0.4854	0.2356	1.1072
RP-8	M	0.2767	0.2442	1.9823	0.5888	0.3533	0.1248	0.6735
	A	0.3712	0.3027	1.1742	0.7299	0.3784	0.1432	0.8348
RP-2	M	0.2822	0.3515	0.7938	0.8476	0.2585	0.0668	0.9695
	A	0.8060	0.3811	−0.4491	0.9191	0.5762	0.3320	1.0512
CN	M	0.3924	0.3006	0.8998	0.7249	0.3990	0.1592	0.8292
	A	0.2958	0.2216	0.7958	0.5344	0.4065	0.1652	0.6113
DIOL	M	0.2401	0.1240	−0.2807	0.2990	0.5422	0.2940	0.3420
	A	0.1506	0.1654	−0.2673	0.3989	0.2904	0.0843	0.4563
NH_2	M	0.1855	0.1389	−0.4491	0.3220	0.4203	0.1766	0.3683
	A	0.4927	0.2273	−1.3290	0.5482	0.5857	0.3430	0.6270
				**ALOGP**				
RP-18	M	0.7924	0.3664	0.9444	0.8836	0.5848	0.3419	1.0107
	A	0.7491	0.1054	0.1408	0.3011	0.9213	0.8488	0.4925
RP-8	M	0.3213	0.1107	1.8004	0.3164	0.6952	0.4832	0.5175
	A	0.4651	0.1150	0.8585	0.3286	0.8031	0.6451	0.5373
RP-2	M	0.4664	0.1482	0.2735	0.4235	0.7237	0.5238	0.6926
	A	0.7496	0.1155	−0.4871	0.3299	0.9077	0.8239	0.5397
CN	M	0.4604	0.1179	0.6437	0.3368	0.7930	0.6289	0.5509
	A	0.3557	0.0803	0.5813	0.2293	0.8280	0.6856	0.3751
DIOL	M	0.0757	0.0834	0.0746	0.2382	0.2895	0.0838	0.3896
	A	0.0655	0.0997	−0.0893	0.2848	0.2141	0.0458	0.4658
NH_2	M	0.1763	0.0640	−0.4672	0.1828	0.6763	0.4574	0.2990
	A	0.3782	0.1073	−1.1537	0.3065	0.7615	0.5799	0.5013
				**MLOGP**				
RP-18	M	0.8262	0.1591	1.0189	0.3808	0.8658	0.7496	0.6234
	A	0.8130	0.1763	0.3109	0.4218	0.8382	0.7026	0.6996
RP-8	M	0.34119	01442	1.8875	0.3449	0.6201	0.3846	0.5648
	A	0.4799	0.1656	1.0157	0.3962	0.6948	0.4828	0.6486
RP-2	M	0.4737	0.2018	0.4469	0.4828	0.6163	0.3798	0.7904
	A	0.8850	0.1442	−0.4662	0.3450	0.8984	0.8071	0.5648
CN	M	0.4514	0.1751	0.8488	0.4189	0.6518	0.4248	0.6858
	A	0.3786	0.1151	0.6776	0.2754	0.7388	0.5459	0.4508
DIOL	M	0.0968	0.0988	0.0612	0.2364	0.3106	0.0965	0.3869
	A	0.0680	0.1196	−0.0680	0.2861	0.1863	0.0347	0.4684
NH_2	M	0.1750	0.0856	−0.3932	0.2049	0.5629	0.3169	0.3355
	A	0.4039	0.1445	−1.0541	0.3457	0.6817	0.4648	0.5659
				**XlogP2**				
RP-18	M	1.1399	0.3409	−0.7286	1.1116	0.7635	0.5830	0.7591

	A	1.1161	0.3688	−1.3896	1.2086	0.7306	0.5338	0.8212
RP-8	M	0.4970	0.2712	1,0855	0.8843	0.5438	0.2957	0.6039
	A	0.6385	0.3055	0.1019	0.9963	0.5942	0.3532	0.6804
RP-2	M	0.6209	0.3779	−0.4279	1.2324	0.5022	0.2523	0.8417
	A	1.1218	0.3949	−2.0467	1.2879	0.7086	0.5021	0.8796
CN	M	0.7203	0.3103	−0.4113	1.0118	0.6344	0.4025	0.6909
	A	0.5161	0.2145	−0.0883	0.6996	0.6479	0.4197	0.4778
DIOL	M	0.2736	0.1676	−0.6152	0.5467	0.4998	0.2498	0.3733
	A	0.3068	0.1925	−0.8720	0.6279	0.4908	0.2409	0.4288
NH_2	M	0.2957	0.1642	−0.8509	0.5353	0.4966	0.2466	0.3656
	A	0.5890	0.2951	−2.0455	0.9623	0.5766	0.3324	0.6572

M – methanol; A – acetone; a – slope, S_a – standard error of the slope, b – intercept, S_b – standard error of the intercept, r, r^2– correlation coefficients, S_e – standard error of the estimate.

Table S8. Terms of the linear correlation according to equations $logP_{calc(1)} = b + a\ logP_{calc\ (2)}$.

$logP_{calc}$ (1)/(2)	a	S_a	b	S_b	r	r^2	S_e
AlogPs/ChemAxon	0.6219	0.1002	1.5929	0.2621	0.9003	0.8104	0.3700
AlogPs/XlogP3	0.6432	0.2672	1.2810	0.7675	0.6258	0.3916	0.6630
AlogPs/AC_logP	0.6430	0.2214	1.6091	0.5334	0.6955	0.4837	0.6108
AlogPs/ALOGP	0.4906	0.0797	1.8452	0.2277	0.8989	0.8080	0.3724
AlogPs/MLOGP	0.6022	0.0824	1.8113	0.1972	0.9251	0.8557	0.3228
AlogPs/XlogP2	0.9309	0.2099	0.1651	0.6845	0.8431	0.7108	0.4675
ChemAxon/XlogP3	0.9111	0.3921	- 0.1598	1.1262	0.6123	0.3744	0.9729
ChemAxon/AC_logP	0.9729	0.3063	0.1640	0.7387	0.7264	0.5285	0.8449
ChemAxon/ALOGP	0.7218	0.1071	0.5724	0.3060	0.9136	0.8346	0.5004
ChemAxon/MLOGP	0.8654	0.1244	0.5655	0.2976	0.9183	0.8432	0.4872
ChemAxon/XlogP2	1.2180	0.3487	- 1.3972	1.1371	0.7771	0.6039	0.7766
XlogP3/AC_logP	0.7158	0.1815	1.1525	0.4378	0.7958	0.6333	0.5007
XlogP3/ALOGP	0.3296	0.1387	1.9536	0.3964	0.6207	0.3853	0.6484
XlogP3/MLOGP	0.4112	0.1605	1.9171	0.3842	0.6492	0.4215	0.6289
XlogP3/XlogP2	0.3851	0.2504	1.7099	0.8164	0.4777	0.2282	0.5575
AC_logP/ALOGP	0.4289	0.1352	1.1976	0.3863	0.7265	0.5278	0.6318
AC_logP/MLOGP	0.5076	0.1627	1.2074	0.3893	0.7208	0.5195	0.6373
AC_logP/XlogP2	0.7081	0.3391	0.0802	1.1057	0.5940	0.3528	0.7551
ALOGP/MLOGP	1.1407	0.1162	0.1117	0.2782	0.9562	0.9144	0.4555
ALOGP/XlogP2	1.7093	0.3460	- 2.8033	1.1283	0.8678	0.7531	0.7705
MLOGP/XlogP2	1.3678	0.2891	- 2.1163	0.9426	0.8583	0.7367	0.6437

a – slope, S_a – standard error of the slope, b – intercept, S_b – standard error of the intercept, r, r^2– correlation coefficients, S_e – standard error of the estimate.
